# Supplementary figures and images for: Care-experienced cHildren and young people’s Interventions to improve Mental health and wEll-being outcomes: Systematic review (CHIMES) protocol
Source: BMJ Open. 2021 Jan 26;11(1):e042815. doi: 10.1136/bmjopen-2020-042815 (PMC7839917; doi:10.1136/bmjopen-2020-042815)

Supplement A: CHIMES Review Logic Model

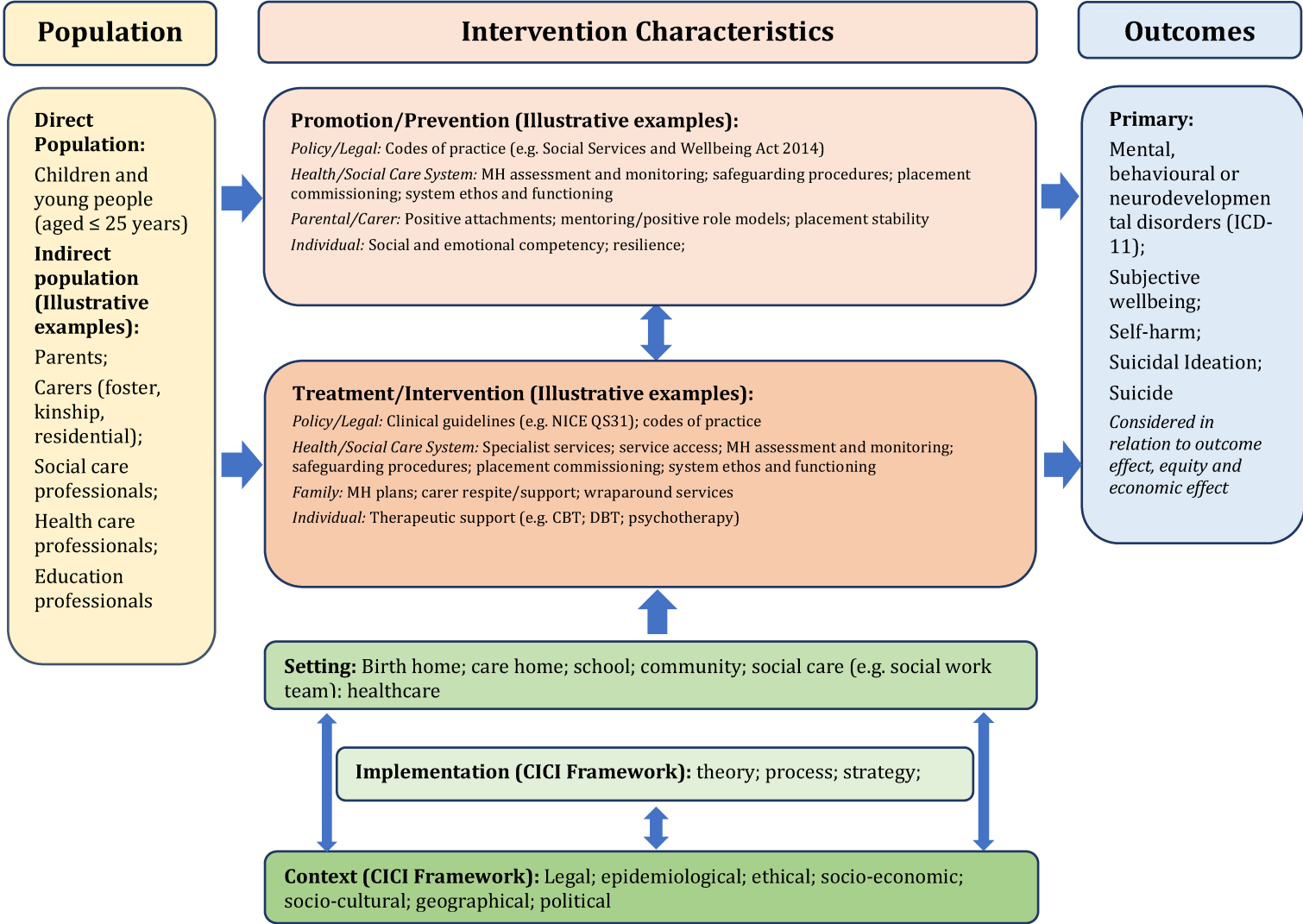

Supplement: Supplementary data [file bmjopen-2020-042815supp001.pdf]
