# Supplementary material for: Care-experienced cHildren and young people’s Interventions to improve Mental health and wEll-being outcomes: Systematic review (CHIMES) protocol
Source: BMJ Open. 2021 Jan 26;11(1):e042815. doi: 10.1136/bmjopen-2020-042815 (PMC7839917; doi:10.1136/bmjopen-2020-042815)
Supplement: Supplementary data [file bmjopen-2020-042815supp002.pdf]

BMJ Publishing Group Limited (BMJ) disclaims all liability and responsibility arising from any reliance placed on this supplemental material which has been supplied by the author(s)

Supplement B: CHIMES Review Search Strategy (Developed in Ovid MEDLINE)

1 exp Child/
2 exp Infant/
3 Young Adult/
4 Adolescent/
5 (teen or teens or teenager*).tw.
6 (adolesc* or preadolesc* or pre-adolesc* or juvenile*).tw.
7 (youth or youths or youngster*).tw.
8 ((young adj (person or persons or people)) or "early adult*").tw.
9 (student or students or schoolchild*).tw.
10 (girl* or boy* or child or children or infant or infants or kid or kids).tw.
11 (pediatri* or paediatric*).tw.
12 (pubescen* or puberty or prepubescen* or pre-pubescen*).tw.
13 orphan*.tw.
14 Child, Foster/
15 Child, Orphaned/
16 "Child of Impaired Parents"/
17 1 or 2 or 3 or 4 or 5 or 6 or 7 or 8 or 9 or 10 or 11 or 12 or 13 or 14 or 15 or 16
18 ((substitute or "local authority" or state or statutory or public or "out of home" or order or place* or group*) adj (care or placement*)).tw.
19 ((nonparent or non-parent) adj3 care).tw.
20 ((children's or childrens) adj home).tw.
21 ((institution* or residential or foster or kinship or group) adj3 (care or home* or placement*)).tw.
22 ("support* living" or "supported lodging*" or "care leaver*").tw.
23 (leaving adj2 care).tw.
24 ((in or welfare or social or respite) adj care).tw.
25 looked after.tw.
26 Special guardian*.tw.
27 Foster Home Care/
28 Child, Institutionalized/
29 18 or 19 or 20 or 21 or 22 or 23 or 24 or 25 or 26 or 27 or 28
30 "Quality of Life"/
31 personal satisfaction/
32 (wellbeing or well-being or "well being").tw.
33 (illbeing or ill-being or "ill being").tw.
34 hedoni*.tw.
35 (eudaimoni* or eudaemoni* or eudemoni*).tw.
36 happiness.tw.
37 ((positive or negative) adj affect).tw.
38 flourish*.tw.
39 ("life satisfaction" or "satisfaction with life").tw.
40 ("positive and negative affect schedule" or PANAS).tw.
41 ("Warwick-Edinburgh Mental Wellbeing" or WEMWBS).tw.
42 ("State Trait Anxiety Inventory" or STAI).tw.
43 "Perceived Stress Scale".tw.
44 SWLS.tw.
45 30 or 31 or 32 or 33 or 34 or 35 or 36 or 37 or 38 or 39 or 40 or 41 or 42 or 43 or 44
46 Mental Health/
47 exp Mental Disorders/
48 Catatonia/
49 Self-Injurious Behavior/
50 Self Mutilation/

51 Suicide/
52 Suicidal Ideation/
53 Suicide, Attempted/
54 Suicide, Completed/
55 "mental health".tw.
56 ("bodily distress" or paraphilic or paraphilia or catatonia or catatonic or dissociation or "impulse control").tw.
57 (schizophrenia or psychotic or psychosis or OCD or "obsessive compulsive disorder").tw.
58 suicid*.tw.
59 (self adj2 (harm or injur* or cutting or mutilation or poison* or burn*)).tw.
60 (("post traumatic" or post-traumatic or posttraumatic) adj2 (stress or disorder*)).tw.
61 ((grief or adjustment or "reactive attachment" or "disinhibited social engagement") adj2 (disorder* or condition* or problem*)).tw.
62 (disruptive adj2 behavio?r*).tw.
63 ((behavio?r* or neurodevelopmental or mood or fear or anxiety or personality or disruptive or dissocial or impulse or factitious or neurocognitive or feeding or eating or elimination or disruptive or dissocial or anxiety or depressive) adj3 (disorder* or condition* or problem*)).tw.
64 46 or 47 or 48 or 49 or 50 or 51 or 52 or 53 or 54 or 55 or 56 or 57 or 58 or 59 or 60 or 61 or 62 or 63
65 45 or 64
66 exp Controlled Clinical Trial/
67 Double-Blind Method/
68 exp "Costs and Cost Analysis"/
69 random allocation/
70 (("pre-test" or pretest or posttest or "post-test" or "pre-intervention" or "post-intervention" or "controlled before" or "before and after" or "follow-up assessment") and (controlled or control or "comparison participants" or "comparison group" or "usual care" or placebo)).tw.
71 ("quasi-experiment*" or quasiexperiment* or "quasi-randomi*" or "quasirandomi*" or "natural* experiment" or "time series" or "interrupted time").tw.
72 ((controlled or control or intervention or comparison) adj3 (group or groups or study or trial or evaluation or cohort or cohorts or longitudinal or matched or matching or experiment or experimental)).tw.
73 ("difference in difference" or "instrumental variable*" or "propensity score matching" or "process evaluation").tw.
74 ((cost or costs or costing or economic) adj1 (analysis or effectiveness or benefit or evaluation or utility or savings or measure or measures)).tw.
75 (trial or "randomi?ed controlled trial" or rct or "cross-over design" or " cross over design" or "crossover design" or "cross-over study" or "cross over study" or "crossover study" or "factorial design" or "controlled study" or "controlled design" or "single-blind" or "single blind" or "double-blind" or "double blind" or "triple-blind" or "triple blind").tw.
76 effectiveness.tw.
77 program evaluation/
78 (program* adj (effect* or efficacy)).tw.
79 ((theor* or mechanism*) adj3 (change or impact or program*)).tw.
80 (("mixed method*" or "mixed-method*" or process or qualitative) adj3 evaluation*).tw.
81 (causal adj2 (assumption* or process*)).tw.
82 66 or 67 or 68 or 69 or 70 or 71 or 72 or 73 or 74 or 75 or 76 or 77 or 78 or 79 or 80 or 81
83 17 and 29 and 65 and 82
