# Supplementary material for: Care-experienced cHildren and young people’s Interventions to improve Mental health and wEll-being outcomes: Systematic review (CHIMES) protocol
Source: BMJ Open. 2021 Jan 26;11(1):e042815. doi: 10.1136/bmjopen-2020-042815 (PMC7839917; doi:10.1136/bmjopen-2020-042815)
Supplement: Author's manuscript [file bmjopen-2020-042815.draft_revisions.pdf]

BMJ Open

BMJ Open is committed to open peer review. As part of this commitment we make the peer review history of every article we publish publicly available.

When an article is published we post the peer reviewers' comments and the authors' responses online. We also post the versions of the paper that were used during peer review. These are the versions that the peer review comments apply to.

The versions of the paper that follow are the versions that were submitted during the peer review process. They are not the versions of record or the final published versions. They should not be cited or distributed as the published version of this manuscript.

BMJ Open is an open access journal and the full, final, typeset and author-corrected version of record of the manuscript is available on our site with no access controls, subscription charges or pay-per-view fees (http://bmjopen.bmj.com).

If you have any questions on BMJ Open's open peer review process please email info.bmjopen@bmj.com

BMJ Open

Care-experienced cHildren and young people's Interventions to improve Mental health and wEllbeing outcomes: Systematic review (CHIMES) Protocol

Journal:	*BMJ Open*
Manuscript ID	bmjopen-2020-042815
Article Type:	Protocol
Date Submitted by the Author:	15-Jul-2020
Complete List of Authors:	Evans, Rhiannon; Cardiff University, DECIPHer, School of Social Sciences<br>Boffey, Maria; The Fostering Network<br>MacDonald, Sarah; Cardiff University, DECIPHer, School of Social Sciences<br>Noyes, Jane; Bangor University, School of Social Sciences<br>Melendez-Torres, G.J; University of Exeter,<br>Morgan, Helen; Cardiff University, SURE, University Library Service<br>Trubey, Robert ; Cardiff University, Centre for Trials Research<br>Robling, Michael; Cardiff University, General Practice<br>Willis, Simone; Cardiff University, Specialist Unit for Review Evidence<br>Wooders, Charlotte; The Fostering Network
Keywords:	PUBLIC HEALTH, MENTAL HEALTH, Child & adolescent psychiatry < PSYCHIATRY, Child protection < PAEDIATRICS

SCHOLARONE™
Manuscripts

I, the Submitting Author has the right to grant and does grant on behalf of all authors of the Work (as defined in the below author licence), an exclusive licence and/or a non-exclusive licence for contributions from authors who are: i) UK Crown employees; ii) where BMJ has agreed a CC-BY licence shall apply, and/or iii) in accordance with the terms applicable for US Federal Government officers or employees acting as part of their official duties; on a worldwide, perpetual, irrevocable, royalty-free basis to BMJ Publishing Group Ltd ("BMJ") its licensees and where the relevant Journal is co-owned by BMJ to the co-owners of the Journal, to publish the Work in this journal and any other BMJ products and to exploit all rights, as set out in our licence.

The Submitting Author accepts and understands that any supply made under these terms is made by BMJ to the Submitting Author unless you are acting as an employee on behalf of your employer or a postgraduate student of an affiliated institution which is paying any applicable article publishing charge ("APC") for Open Access articles. Where the Submitting Author wishes to make the Work available on an Open Access basis (and intends to pay the relevant APC), the terms of reuse of such Open Access shall be governed by a Creative Commons licence – details of these licences and which Creative Commons licence will apply to this Work are set out in our licence referred to above.

Other than as permitted in any relevant BMJ Author's Self Archiving Policies, I confirm this Work has not been accepted for publication elsewhere, is not being considered for publication elsewhere and does not duplicate material already published. I confirm all authors consent to publication of this Work and authorise the granting of this licence.

For peer review only

Care-experienced cHildren and young people's Interventions to improve Mental health and wEllbeing outcomes: Systematic review (CHIMES) Protocol

Dr Rhiannon Evans[1]
Corresponding Author
Email: EvansRE8@cardiff.ac.uk
Tel: 02920 870099

Maria Boffey[2] Email: Maria.Boffey@fostering.net

Dr Sarah MacDonald[1] Email: MacDonaldS@cardiff.ac.uk

Prof Jane Noyes[3] Email: jane.noyes@bangor.ac.uk

Prof GJ Melendez Torres[4] Email: g.j.melendez-torres@exeter.ac.uk

Dr Helen Morgan[5] Email: MorganHE1@cardiff.ac.uk

Dr Rob Trubey[6] Email: TrubeyRJ@cardiff.ac.uk

Prof Michael Robling[6] Email: RoblingMR@cardiff.ac.uk

Simone Willis[5] Email: WillisS5@cardiff.ac.uk

Charlotte Wooders[2] Email: Charlotte.Wooders@fostering.net

[1]DECIPHer, School of Social Sciences, Cardiff University, 1-3 Museum Place, Cardiff, CF10 3BD
[2]The Fostering Network, 33 Cathedral Road, Cardiff, CF11 9HB
[3] School of Health Sciences, Bangor University, Bangor, LL57 2DG
[4]Peninsula Technology Assessment Group (PenTAG), University of Exeter, South Cloisters, St Luke's Campus, Exeter, EX1 2LU.
[5]Specialist Unit for Review Evidence, Cardiff University, Neuadd Meirionnydd, Heath Park Campus, Cardiff, CF14 4YS
[6] Centre for Trials Research, Cardiff University, Neuadd Meirionnydd, Heath Park Campus, Cardiff, CF14 4YS

For peer review only

Abstract

Introduction: The mental health and wellbeing of children and young people who have been in care (i.e. care-experienced) is a priority. There are a range of interventions aimed at addressing these outcomes, but the international evidence-base remains ambiguous. There is a paucity of methodologically robust systematic reviews of intervention effectiveness, with few considering the contextual conditions under which evaluations were conducted. This is important in understanding the potential transferability of the evidence-base across contexts. The present systematic review will adopt a complex systems perspective to synthesise evidence reporting evaluations of mental health and wellbeing interventions for care-experienced children and young people. It will address impact, equity, cost-effectiveness, context, implementation and acceptability. Stakeholder consultation will prioritise a programme theory, and associated intervention, that may progress to further development and evaluation in the UK.

Methods and Analysis: We will search 15 bibliographic databases from 1990-June 2020. Supplementary searching will include citation tracking, author recommendation, and identification of evidence clusters relevant to included evaluations. The eligible population is children and young people (aged ≤ 25 years) with experience of being in care. Outcomes are 1) mental, behavioural or neurodevelopmental disorders; 2) subjective wellbeing; 3) self-harm; suicidal ideation; suicide. Study quality will be appraised with methodologically appropriate tools. We will construct a taxonomy of programme theories and intervention types. Thematic synthesis will be used for qualitative data reporting context, implementation and acceptability. If appropriate, meta-analysis will be conducted with outcome and economic data. Convergent syntheses will be used to integrate syntheses of qualitative and quantitative data.

Ethics and Dissemination: We have a comprehensive strategy for engagement with care-experienced children and young people, carers and social care professionals. Dissemination will include academic and non-academic publications and conference presentations. Ethical approval from Cardiff University's School of Social Sciences REC will be obtained if necessary.

PROSPERO Registration: CRD42020177478

Strengths and limitations of this study

- This review will offer one of the first methodologically robust syntheses of evidence reporting evaluations of mental health and wellbeing interventions for care-experienced children and young people;
- In taking a complex systems approach, the review will provide insights into the potential transferability of interventions and the replicability of effects across contexts;
- From this review, the study team aim to identify a potential programme theory (and associated intervention) that can progress to further development and evaluation in the UK context;
- The review will support policymakers and practitioners in decision-making about how to support the mental health and wellbeing of children and young people who have been in care;
- The review is limited by only including studies published in higher resource countries.

For peer review only

Background

Rationale

The mental health and wellbeing of children and young people who have been in care (i.e. care-experienced) is a growing public health and social care concern. Internationally, almost 50% of individuals involved in the child welfare system have a diagnosable mental health condition (1). Meanwhile in the UK, they are nearly five times as likely to have at least one psychiatric diagnosis compared to the general population (2). Care-experienced individuals are at an elevated risk of poor subjective wellbeing (3) and are more than four times as likely to attempt suicide (3). Adverse mental health outcomes incur significant health and social care costs, often due to the associated risk of placement instability and breakdown (4, 5). Evidence from the UK reports that insecure and unstable care placements cost £22,415 more per child per annum (across health, social care and criminal justice) than stable care pathways (6).

There is a clear need to improve mental health provision for those who experience care, with NICE guidance stating that the UK evidence base does not adequately serve this population (7). Equally, while the Department for Education and Department of Health and Social Care's joint statutory guidance has mandated that local authorities ensure timely and adequate services (8), there are reported incidents of failure to identify need, overly stringent eligibility thresholds, and withholding of care where there is not a stable placement (9). A comprehensive synthesis is required to identify evidence-based interventions in order to develop recommendations to enhance policy and practice.

Internationally there is a large evidence base for interventions (10-21), although in the UK it remains emergent. Current interventions focus on preventative approaches that operate across socio-ecological domains, including community level change (e.g. Flying Start) (22), functioning within the care placement (23, 24), the theoretical orientation of social care teams (e.g. adoption of a trauma informed model) (25), and the social and emotional competencies of the individual child or young person (26). Meanwhile, treatment has tended to focus on both the type and availability of therapeutic approaches, such as counselling or access to mental health services (27).

Despite the size of the extant literature, there are limitations that need to be attended to, notably in regard to existing systematic reviews. First, the most inclusive syntheses lack rigorous methodologies. One of the most recent and relevant literature reviews recognised that it is not exhaustive (10). Second, reviews tend to focus on a limited number of intervention types (e.g. Treatment Foster Care) (11), populations (e.g. foster care) (11, 13, 17, 26, 27), or outcomes (e.g. externalising behaviours) (17, 18). This makes it difficult to know if a particular

For peer review only

intervention can work effectively with different populations across multiple outcomes in order to reduce the number of approaches needing to be resourced within a system. Third, reviews often include only rudimentary treatment of context and process data. Understanding the complex system in which interventions operate is imperative. International variations in social care and health care systems may constrain the transferability of evidence and the extent to which we can 'borrow strength' from the original evaluation (28). For example, Multi-systematic therapy (MST) is shown to not be effective in Sweden, despite demonstrating effects in the USA, as it is essentially equal to usual care (29).

The present mixed method systematic review will use a robust methodology to draw together the evidence-base for interventions aimed at improving the mental health and wellbeing of care-experienced children and young people, including those currently in care and those who have previously been in care. Adopting a complex systems perspective (30, 31), we will synthesise evidence of impact, equity and cost effectiveness, while also focusing on understanding how intervention effects are contingent on the context in which they are evaluated. In collaboration with stakeholders (young people, carers and social care professionals), the resulting synthesis will be used to identify, where possible, an overarching programme theory (or theories) that may inform the development or adaptation of an intervention to progress to further evaluation within the UK context.

Review Aims and Questions

The study aims to systematically review and synthesise international evidence on interventions to improve the mental health and wellbeing of care-experienced children and young people. We will address the following research questions:

RQ1: What are the types, theories and outcomes tested in mental health and wellbeing interventions for care-experienced children and young people?

RQ2: What are the effects (including inequities and harms) and economic effects of interventions?

RQ3: How do contextual characteristics shape implementation factors, and what are key enablers and inhibitors of implementation?

RQ4: What is the acceptability of interventions to key stakeholders?

RQ5: Can and how might intervention types, theories, components, and outcomes be related in an overarching system-based programme theory?

For peer review only

RQ6: Drawing on the findings from RQ1-5, what do stakeholders think is the most effective, feasible and acceptable intervention in the UK that could progress to further outcome or implementation evaluation?

Methods

Protocol and Registration

The protocol is registered with PROSPERO and reported in accordance with the Preferred Reporting Items for Systematic reviews and Meta-Analyses Protocols (PRISMA-P) guidelines (32). The review will be published in accord with the Preferred Reporting Items for Systematic reviews and Meta-Analyses (33), with qualitative synthesis being structured by the ENTREQ reporting standards (34) and the mixed methods process evaluation synthesis following the principles prescribed by Cochrane (35).

Eligibility Criteria

The eligibility parameters are reported in accordance with the PICOS framework and presented in the logic model (Supplement A).

Population: Children and young people aged $\leq$ 25 year olds. Currently placed in care or previous care experience. Period of time in care is not restricted. Care can include in-home care (i.e. voluntary transfer of parental responsibility to statutory services) and out-of-home care (e.g. foster care, residential care, or formal kinship care). The following are excluded: general population; children in need; individuals at the edge of care; care without specified statutory involvement (e.g. informal kinship care); adoption; and refugees. Any population may be targeted as intervention participants (e.g. carer, birth family, teacher or social worker) but outcomes must be reported at the level of the child or young person.

Intervention: Any attempt to disrupt existing system practices. They may be mono-component or multi-component and operate across any of the following socio-ecological domains: individual; inter-personal; organisational; community; and policy/legal. Interventions may focus on promotion/prevention or the management/treatment of symptomology. There are no a-priori criteria for implementation (i.e. delivery setting, delivery mode, delivery agent). The following are excluded: pharmacological interventions.

Comparator: Treatment as usual; other active treatment; no specified treatment.

Outcomes: Three domains of primary outcomes: 1) Mental, behavioural or neurodevelopmental disorders as specified by ICD 11; 2) Subjective wellbeing (eudaimonia and hedonia); 3) Self-harm; suicidal ideation; suicide. Measures may use dichotomous, categorical or continuous

For peer review only - http://bmjopen.bmj.com/site/about/guidelines.xhtml

variables. Outcomes may be ascertained through clinical assessment, self-report or report by another informant (e.g. teacher).

Study Design: Programme theory: Describe intended theory, logic model or mechanisms of effect. May include mediation analysis (RQ1); *Outcome evaluation:* (Individual/cluster) randomised controlled trials and quasi-experimental study designs. We exclude post measurement only or pre-post measurement in intervention group only (RQ1; RQ2); *Process evaluation:* All qualitative and quantitative study designs (RQ1; RQ3; RQ4). Included studies must empirically report on relevant contextual influences, implementation or acceptability; *Economic evaluation*: Must relate costs to benefits and can report: cost-minimisation, cost-effectiveness, cost utility or cost-benefit analysis. May be model-based or trial-based. Decision-analytic models capturing intervention impacts on mental health and wellbeing will be eligible (RQ2). *Relationship Between Study Designs:* To be included programme theory papers must have an associated empirical outcome evaluation. Process evaluations and economic evaluations do not necessarily have to be linked to an empirical outcome evaluation, as they provide wider contextual insight into how interventions interact with complex system characteristics.

Information Sources

The following electronic bibliographic databases will be searched: ASSIA, British Education Index, Child Development & Adolescent Studies, CINAHL, Embase, ERIC, Cochrane Database of Clinical Controlled Trials (CENTRAL), HMIC, IBSS, Medline (Medline in Process and Medline ePub), PsycINFO, Scopus, Social Policy & Practice, Social Services Abstracts, Sociological Abstracts and Web of Science (Social Sciences Citation Index, Conference Proceedings Citation Index- Social Science & Humanities, Emerging Sources Citation Index). Supplementary searching techniques will include citation tracking of included studies, contacting international experts, searching trial registers and consulting websites of key social and health care organisations. Searches will be conducted for 'clusters' of reports related to included studies to develop descriptions of programme theories and further progress understanding of context (36, 37). Inclusion will be restricted from 1990, which marked the ratification of the UN Convention on the Rights of the Child (38). This prescribed comprehensive social and health care provision for children internationally and started the proliferation of intervention in this area. To maximise applicability of evidence to the UK, studies from lower-middle income countries will be excluded. All languages will be included in searches.

Search Strategy

We developed a provisional search strategy in Ovid MEDLINE (Supplement B). It will be adapted to the functionality of each database on study commencement.

For peer review only

Data Management and Study Selection

Data will be exported to Endnote for de-duplication and then to EPPI Reviewer Web for screening. In the first instance reference titles will be screened on the basis of title to identity clearly irrelevant retrievals (e.g. pharmacological treatments), with these exclusions being verified by a second reviewer. Titles and abstracts of remaining studies will be screened independently and in duplicate. The inclusion criteria pro-forma will be piloted and calibrated by two reviewers screening the same 100 title and abstracts, with disagreement being resolved through consensus or recourse to a third reviewer. Full texts will be retrieved and appraised for study inclusion. Conflicts will be resolved through discussion or recourse to a third reviewer. A record of the selection process will be retained in adherence with the PRISMA flow diagram.

Data Collection

Once the final number of included studies is confirmed, we will chart clusters of studies, constructing a visual knowledge map similar to that recommended within scoping reviews (39, 40). This process will support decision-making about the depth of data extraction at the next stage (e.g. sampling may be conducted for qualitative studies) and extent of synthesis to be undertaken (e.g. meta-analysis may be conducted where there are multiple studies). The CHIMES Review has a Study Advisory Group that will be consulted at this stage to confirm the extraction and synthesis.

Standardised data extraction forms will be developed and calibrated with a subset of studies for input to EPPI Reviewer Web. Two versions of extraction forms may be developed based on the mapping of the data. The first will be for studies included in the review but not included in more in-depth synthesis, and the second for studies subjected to more in-depth synthesis. For primary qualitative studies a subset of data will be coded in vivo to develop a preliminary coding tree with a priori codes, but which can be amended to incorporate emergent codes. On confirmation of the extraction forms and coding tree, two reviewers will independently extract and code data from 10% of studies, with the remainder being extracted by one reviewer and verified by a second.

Data Items

For all studies data will be extracted on: study characteristics, participant demographics; setting; study design and methods; intervention theory of change and intervention characteristics. For outcome evaluations data will be extracted for: measurements, sequence generation, allocation concealment, blinding, data completeness, baseline differences and adjustment for difference, control of confounding, and outcomes at follow-up at both population and sub-group level. Data items from process evaluations will be the implementation theory,

For peer review only

implementation strategy and implementation agents. Quantifiable assessments of implementation will be reach, receipt, and fidelity. Contextual characteristics will be classified according to the CICI framework: geographical; epidemiological; legal; socio-economic; socio-cultural; ethical; and political (41). Acceptability data across stakeholders will be extracted. Economic evaluation data items will be intervention and comparator costs, perspective, structural and empirical inputs, time horizon, and cost-effectiveness. Where there is incomplete data the author will be asked for additional information and where this is not retrieved the data will be reported as missing and included in the risk of bias assessment.

Risk of Bias (Individual Studies)

Study data will be appraised with a methodologically appropriate tool. Programme theory will be appraised through an assessment of clarity of constructs, clarity of relationships between constructs, testability, parsimony and generalisability (42). Outcome evaluations will be assessed with the relevant tool prescribed by the Cochrane Handbook for Systematic Reviews of Interventions (43). Qualitative data within process evaluations will be appraised using a tool developed in previous systematic reviews, assessing reliability and usefulness (44, 45). Economic data will be appraised with the Drummond checklist (46). We will also consider the governance and ethical conduct of studies (47). All quality appraisal will be undertaken independently and in duplicate, with disagreement being resolved through discussion or recourse to a third reviewer.

Synthesis of Results

Programme Theory, Context, Implementation and Acceptability (RQ1; RQ4; RQ5). We will construct a single taxonomy describing intervention types, theories and outcomes. It will be used to understand if there are different or dominant theories according to different types of interventions and/or outcomes (42). Context and implementation will be classified according to the CICI framework (41). Implementation will also comprise data on quantifiable assessments of implementation, including: reach; receipt; and fidelity. Acceptability will be considered as the experiences of all stakeholders, including participants, delivery agents and service funders, and their interactions with the interventions.

Analysis will adhere to the five phases of framework synthesis (48-51): 1) Familiarisation with included studies; 2) Develop a thematic framework based on the parameters of the review. This will be refined and calibrated with a subset of data; 3) Index the remaining corpus of data. New themes, and associated codes, may be generated in vivo through the process of constant comparison across studies and against the framework; 4) Charting the data into a framework matrix summarizing data by category from each transcript.; 5) Mapping and interpreting data to

For peer review only

create the typology of interventions and to explain associations between themes to help understand how and why interventions may or may not be effective. The synthesis may be presented graphically and narratively.

Intervention Outcome Effects, Equities and Economic Effects (RQ2). The taxonomy describing intervention types, theories and outcomes will inform and structure the analyses undertaken as part of the quantitative synthesis. Depending on intervention heterogeneity we will consider meta-analysing effect estimates from outcome evaluations. In preparation for meta-analysis, we will extract effect estimates from studies that will be classified into outcome domains. Where appropriate outcomes will be converted to odds ratios using logistic transformation for pooling. Estimates from cluster randomised trials will be checked for unit of analysis issues, and where necessary, an inflation factor will be applied to the standard error of effect estimates. Where intra-cluster correlation coefficients are not available and effect estimates have not been adjusted for clustering, we will impute an ICC using the average of estimates for specific outcomes from 'most similar' intervention evaluations.

We will undertake robust variance estimation meta-analyses according to intervention type, outcome and timepoint, considering up to six months from baseline as short-term, six months to two years as mid-term, and beyond two years as long-term. Robust variance estimation meta-analysis is a method that permits the inclusion of more than one effect estimate per study in a meta-analysis; this is in contrast to standard meta-analysis models that assume independence between individual effect estimates. It is common in meta-analysis of psychosocial interventions for outcome evaluations to present multiple relevant effect estimates per outcome (e.g. multiple estimates of child behavioural problems). This method will permit use of all relevant information from included studies. Within each meta-analysis, we will examine heterogeneity using a combination of Cochran's Q, tau-squared and I^2. Where heterogeneity is substantial (I^2 >50%), we will scrutinise included studies to hypothesise and explore the reasons for this.

Equity effects will be categorized according to PROGRESS-Plus (52). Data will be reported in adherence to the PRISMA E-2012 extension (53). We will use harvest plots to assess equity effects and meta-regression to test whether characteristics of study populations are associated with effectiveness. Intervention harms will be treated in accordance with the PRISMA harms extension (54).

Economic evaluations will be summarized. Summarized data will include measures of costs, cost -effectiveness, indirect resource use, and whether a trial-based or model-based analysis was conducted. If there is sufficient homogeneity in measures across studies, these will be synthesised via meta-analysis. Measures of costs, cost-effectiveness and indirect resource use

For peer review only

will be adjusted in line with inflation and currency to provide a contextually relevant estimate of costs in the current UK context.

Synthesis of qualitative and quantitative syntheses: The review will adhere to a convergent synthesis design (55, 56). This will entail the separate synthesis and reporting of qualitative and quantitative data in methodologically relevant but complementary manner, before integrating them in a further synthesis (Figure 1). At this stage the synthesis of context, implementation and acceptability will be integrated with outcome data to explain intervention effectiveness and potential inequities. To support this we will use a narrative summary and a 2x2 matrix that will map context, implementation, acceptability, participant values and costs against the included intervention theories/types (57). The matrix can demonstrate where an intervention may have (in)sufficiently accommodated factors that led to its (in)effectiveness, and what factors need to be better attended to moving forward. We will additionally develop a logic model(s) of a potentially effective programme theory or theories that is a best fit with the evidence base.

To support harmonisation and integration of syntheses across the review, we have formulated research questions that are complementary and contingent, where achieving a comprehensive answer to one question is dependent on the answers to other questions. When conducting the review, we will: 1) Have the same members of the research team work on both the synthesis of qualitative and quantitative data; 2) Screen all study types simultaneously and by the same members of the research team, storing data in EPPI Reviewer to ensure easy sharing of study data across syntheses; and 3) Use method specific appraisal tools that have been combined in previous reviews due to providing epistemological flexibility or consonance. We will consider appraising the certainty of evidence generated through the different reviews with the compatible tools of GRADE (58) and GRADE CERQual (59).

Programme Theory Modelling, Stakeholder Consultation and Intervention Prioritisation

From the review findings, we will aim to identify an overarching programme theory (or compatible programme theories across socio-ecological domains), and associated interventions, which can address multiple outcomes across a number of populations. These will be diagrammatically depicted with the use of system-based logic models and an accompanying narrative summary (60). Stakeholder consultations will be held to prioritise the theories and interventions that might be most feasible and acceptable within the UK context and which may progress to further evaluation (See PPI section for stakeholder description). There are three phases of assessment as part of this prioritisation process (Figure 2):

For peer review only

Phase 1 Intervention Identification: Stakeholders will assess and rank candidate programme theories, and associated interventions, against the following progression criteria: 1) acceptability; 2) feasibility (particularly in UK context); and 3) perceived effectiveness. At the end of this phase there should be a potential programme theory and possible intervention components. Where no candidate theories meet the progression criteria, or an overarching theory is identified but there are no intervention components (e.g. no intervention embodies all of the interacting aspects of the theory), we will consider that de novo intervention development is required.

Phase 2 Intervention Development and Adaptation: If a programme theory is identified, and has an associated intervention, stakeholders will assess if adaptation is required for the UK context (if evaluated elsewhere). This will be done by mapping similarities in contexts using a simplified version of the CICI framework (41). If contexts are considered dissimilar, stakeholders will consider the types of adaptations that may be required. In progress MRC-NIHR funded guidance on adaptation will support this discussion (61). If a de novo intervention is deemed necessary, we will ask stakeholders to undertake preliminary consultation on what an intervention might look like, considering what contextual factors need to be accommodated in order to make it acceptable, feasible and effective.

Phase 3 Intervention Evaluation: This phase will consider the appropriate research design for progressing the intervention. We will use the Medical Research Council's guidance on developing and evaluating complex interventions to define the phase of evaluation (62). To support decision making in the case where it is appropriate to evaluate an existing intervention (adapted or non-adapted), we will use the 'borrowing strength' framework to assess if any outcome data from the original context is applicable within the UK context (28). The framework dictates that if contexts are largely congruent or adaptations are minimal, then an implementation study may be warranted. Where contexts are significantly dissimilar or substantial adaptation has been undertaken, more extensive feasibility and outcome evaluation will be required. It is likely that decision-making at this phase will be predominantly conducted by the research team. On completion of the three prioritisation phases, we anticipate having a clearly identified research agenda to progress beyond the review.

Ethics and Dissemination

Ethics

Cardiff University's School of Social Sciences Research Ethics Committee will provide ethical approval for the stakeholder consultation if required.

Patient and Public Involvement

For peer review only

The review includes a comprehensive programme of patient and public (PPI) involvement. Engagement with children and young people will be facilitated through CASCADE Voices, a collaboration between Voices from Care Cymru and the Children's Social Care Research and Development Centre (CASCADE) at Cardiff University. CASCADE Voices comprises a research advisory group of care-experienced young people. We will consult with them at study commencement to refine and confirm the scope and focus of the review, notably the key subgroups to be analysed and the contextual influences on implementation.

To support the prioritisation of interventions moving forward, we will hold a consultation with CASCADE Voices. A social care practitioner workshop will be hosted with the ExChange Wales Network. The network, hosted by CASCADE, brings together social care sector stakeholders to share experiences and expertise. Further consultation opportunities will be sought with the UKRI funded Transdisciplinary Research for the Improvement of Young Mental Public Health (TRIUMPH) Network. THE CHIMES study Principal Investigator (RE) co-leads the Networks' programme on the mental health of key groups, which includes care-experienced young people.

Dissemination

Outputs from the study will be 1) NIHR PHR monograph; 2) academic publications reporting the results; and 3) a briefing report for policy and practitioners. Presentations will be delivered at academic, policy and practice workshops and conferences, both nationally and internationally. Networks and research infrastructures will be utilised to disseminate findings, notably TRIUMPH and the What Works Centre for Children's Social Care. The latter organisation was funded by the Department for Education and established in collaboration with Cardiff University, with the aim of improving outcomes for children in care. It has a focus on evidence synthesis, providing a depository of evidence summaries on their website for social care professionals. Further dissemination activities will be identified in collaboration with PPI stakeholders throughout the course of the study.

Conclusion

The CHIMES review addresses a significant evidence gap within social and health care, namely which interventions are most likely to be impactful, equitable, cost-effective, feasible and acceptable in improving the mental health and wellbeing of care-experienced children and young people. In conducting a review with methodological robustness and a comprehensive remit, no further synthesis should be required in the short to medium term, ensuring that resources can be invested in intervention development, evaluation and implementation. Adoption of a complex systems lens should support the delivery of more effective interventions, as they are responsive to local needs and anticipate system inhibitors of implementation. Most

For peer review only

fundamentally, in developing knowledge of which interventions are likely to work, finite public resources can hopefully be expended on approaches that can best ensure the positive mental health and wellbeing of care-experienced children and young people, while encouraging disinvestment from ineffective and potentially harmful approaches.

For peer review only

References

1. Bronsard G, Alessandrini M, Fond G, Loundou A, Auquier P, Tordjman S, et al. The Prevalence of Mental Disorders Among Children and Adolescents in the Child Welfare System: A Systematic Review and Meta-Analysis. Medicine. 2016;95(7):e2622-e.
2. Ford T, Vostanis P, Meltzer H, Goodman R. Psychiatric disorder among British children looked after by local authorities: Comparison with children living in private households. British Journal of Psychiatry. 2018;190(4):319-25.
3. Long SJ, Evans RE, Fletcher A, Hewitt G, Murphy S, Young H, et al. Comparison of substance use, subjective well-being and interpersonal relationships among young people in foster care and private households: a cross sectional analysis of the School Health Research Network survey in Wales. BMJ Open. 2017;7(2).
4. James S. Why Do Foster Care Placements Disrupt? An Investigation of Reasons for Placement Change in Foster Care. Social Service Review. 2004;78(4):601-27.
5. Rubin DM, Alessandrini EA, Feudtner C, Mandell DS, Localio AR, Hadley T. Placement Stability and Mental Health Costs for Children in Foster Care. Pediatrics. 2004;113(5):1336-41.
6. Curtis L, Burns A. Unit Costs of Health and Social Care 2017. Canterbury: Personal Social Services Research Unit, University of Kent; 2017.
7. NICE. Looked-after children and young people. Public health guideline [PH28]. London: National Institute for Health and Care Excellence; 2010.
8. Department for Education. Promoting the health and well-being of looked-after children. Statutory guidance for local authorities, clinical commissioning groups and NHS England. London: Department for Education; 2015.
9. House of Commons Education Committee. Mental health and well-being of looked-after children. Fourth Report of Session 2015–16. London: House of Commons Education Committee; 2016.
10. Luke N, Sinclair I, Woolgar M, Sebba J. What works in preventing and treating poor mental health in looked after children. London: NSPCC. 2014.
11. Turner W, Macdonald G. Treatment foster care for improving outcomes in children and young people: A systematic review. Research on Social Work Practice. 2011;21(5):501-27.
12. Everson-Hock ES, Jones R, Guillaume L, Clapton J, Goyder E, Chilcott J, et al. The effectiveness of training and support for carers and other professionals on the physical and emotional health and well-being of looked-after children and young people: a systematic review. Child Care Health Dev. 2012;38(2):162-74.
13. Hambrick EP, Oppenheim-Weller S, N'Zi A M, Taussig HN. Mental Health Interventions for Children in Foster Care: A Systematic Review. Child Youth Serv Rev. 2016;70:65-77.
14. Everson-Hock ES, Jones R, Guillaume L, Clapton J, Duenas A, Goyder E, et al. Supporting the transition of looked-after young people to independent living: a systematic review of interventions and adult outcomes. Child Care Health Dev. 2011;37(6):767-79.
15. Kerr L, Cossar J. Attachment Interventions with Foster and Adoptive Parents: A Systematic Review. Child Abuse Review. 2014;23(6):426-39.
16. O'Higgins A, Ott EM, Shea MW. What is the Impact of Placement Type on Educational and Health Outcomes of Unaccompanied Refugee Minors? A Systematic Review of the Evidence. Clinical child and family psychology review. 2018;21(3):354-65.
17. Marsh D. A systematic review and meta-analysis of randomized clinical trials of evidence-based practices through measured change of behavior for children in foster care 2017.
18. Solomon DT, Niec LN, Schoonover CE. The Impact of Foster Parent Training on Parenting Skills and Child Disruptive Behavior. Child Maltreat. 2017;22(1):3-13.
19. Sullivan AL, Simonson GR. A systematic review of school-based social-emotional interventions for refugee and war-traumatized youth. Review of Educational Research. 2016;86(2):503-30.

For peer review only

20. Macdonald GM, Turner W. Treatment foster care for improving outcomes in children and young people. The Cochrane database of systematic reviews. 2008(1):Cd005649.
21. Dickson K, Sutcliffe K, Gough D, Statham J. Improving the emotional and behavioural health of looked-after children and young people (LACYP): research review 2. London: Centre for Excellence and Outcomes in Children and Young People's Services (C4EO); 2009.
22. Hutchings J, Griffith N, Bywater T, Williams ME. Evaluating the Incredible Years Toddler Parenting Programme with parents of toddlers in disadvantaged (Flying Start) areas of Wales. Child: Care, Health and Development. 2017;43(1):104-13.
23. Sinclair I, Parry E, Biehal N, Fresen J, Kay C, Scott S, et al. Multi-dimensional Treatment Foster Care in England: differential effects by level of initial antisocial behaviour. European Child & Adolescent Psychiatry. 2016;25(8):843-52.
24. Briskman J, Castle J, Blackeby K, Bengo C, Slack K, Stebbens C, et al. Randomised controlled trial of the fostering changes programme. London: National Academy for Parenting Research/London: King's College/Department of Education. 2012.
25. Lang JM, Campbell K, Shanley P, Crusto CA, Connell CM. Building Capacity for Trauma-Informed Care in the Child Welfare System:Initial Results of a Statewide Implementation. Child Maltreatment. 2016;21(2):113-24.
26. Taussig HN, Culhane SE. Impact of a Mentoring and Skills Group Program on Mental Health Outcomes for Maltreated Children in Foster CareMentoring/Skills Group Program and Foster Care. JAMA Pediatrics. 2010;164(8):739-46.
27. Bellamy JL, Gopalan G, Traube DE. A national study of the impact of outpatient mental health services for children in long-term foster care. Clinical Child Psychology and Psychiatry. 2010;15(4):467-79.
28. Aarons GA, Sklar M, Mustanski B, Benbow N, Brown CH. "Scaling-out" evidence-based interventions to new populations or new health care delivery systems. Implementation Science. 2017;12(1):111.
29. Westermark PK, Hansson K, Olsson M. Multidimensional treatment foster care (MTFC): results from an independent replication. Journal of Family Therapy. 2011;33(1):20-41.
30. Petticrew M, Knai C, Thomas J, Rehfuess EA, Noyes J, Gerhardus A, et al. Implications of a complexity perspective for systematic reviews and guideline development in health decision making. BMJ Global Health. 2019;4(Suppl 1):e000899.
31. Booth A, Moore G, Flemming K, Garside R, Rollins N, Tunçalp Ö, et al. Taking account of context in systematic reviews and guidelines considering a complexity perspective. BMJ Global Health. 2019;4(Suppl 1):e000840.
32. Moher D, Shamseer L, Clarke M, Ghersi D, Liberati A, Petticrew M, et al. Preferred reporting items for systematic review and meta-analysis protocols (PRISMA-P) 2015 statement. Syst Rev. 2015;4(1):1.
33. Moher D, Liberati A, Tetzlaff J, Altman DG. Preferred reporting items for systematic reviews and meta-analyses: the PRISMA statement. PLoS medicine. 2009;6(7):e1000097.
34. Tong A, Flemming K, McInnes E, Oliver S, Craig J. Enhancing transparency in reporting the synthesis of qualitative research: ENTREQ. BMC Medical Research Methodology. 2012;12(1):181.
35. Flemming K, Booth A, Hannes K, Cargo M, Noyes J. Cochrane Qualitative and Implementation Methods Group guidance series-paper 6: reporting guidelines for qualitative, implementation, and process evaluation evidence syntheses. J Clin Epidemiol. 2018;97:79-85.
36. Harris JL, Booth A, Cargo M, Hannes K, Harden A, Flemming K, et al. Cochrane Qualitative and Implementation Methods Group guidance series-paper 2: methods for question formulation, searching, and protocol development for qualitative evidence synthesis. J Clin Epidemiol. 2018;97:39-48.
37. Booth A, Harris J, Croot E, Springett J, Campbell F, Wilkins E. Towards a methodology for cluster searching to provide conceptual and contextual "richness" for systematic reviews of complex interventions: case study (CLUSTER). BMC Medical Research Methodology. 2013;13(1):118.

For peer review only

38. UNICEF. The United Nations Convention on the Rights of the Child. London: UNICEF; 1989.
39. Arksey H, O'Malley L. Scoping studies: towards a methodological framework. International journal of social research methodology. 2005;8(1):19-32.
40. Levac D, Colquhoun H, O'Brien KK. Scoping studies: advancing the methodology. Implementation science. 2010;5(1):69.
41. Pfadenhauer LM, Gerhardus A, Mozygemba K, Lysdahl KB, Booth A, Hofmann B, et al. Making sense of complexity in context and implementation: the Context and Implementation of Complex Interventions (CICI) framework. Implementation Science. 2017;12(1):21.
42. Bonell C, Hinds K, Dickson K, Thomas J, Fletcher A, Murphy S, et al. What is positive youth development and how might it reduce substance use and violence? A systematic review and synthesis of theoretical literature. BMC public health. 2015;16(1):135.
43. Higgins JP, Green S. Cochrane handbook for systematic reviews of interventions: John Wiley & Sons; 2011.
44. Rees R, Oliver K, Woodman J, Thomas J. The views of young children in the UK about obesity, body size, shape and weight: a systematic review. BMC Public Health. 2011;11:188.
45. Shepherd J, Harden A, Rees R, Brunton G, Garcia J, Oliver S, et al. Young people and healthy eating: a systematic review of research on barriers and facilitators. Health Education Research. 2005;21(2):239-57.
46. Drummond MF, Sculpher MJ, Claxton K, Stoodart GL, Torrance G. Methods for the Economic Evaluation of Health Care Programmes. USA: Oxford University Press; 2015.
47. Vergnes J-N, Marchal-Sixou C, Nabet C, Maret D, Hamel O. Ethics in systematic reviews. Journal of Medical Ethics. 2010;36(12):771-4.
48. Oliver SR, Rees RW, Clarke-Jones L, Milne R, Oakley AR, Gabbay J, et al. A multidimensional conceptual framework for analysing public involvement in health services research. Health expectations : an international journal of public participation in health care and health policy. 2008;11(1):72-84.
49. Brunton G, Oliver S, Oliver S, Lorenc T. A synthesis of research addressing children's, young people's and parents' views of walking and cycling for transport. London: Eppi Centre; 2006.
50. Barnett-Page E, Thomas J. Methods for the synthesis of qualitative research: a critical review. BMC Medical Research Methodology. 2009;9(1):59.
51. Gale NK, Heath G, Cameron E, Rashid S, Redwood S. Using the framework method for the analysis of qualitative data in multi-disciplinary health research. BMC medical research methodology. 2013;13:117-.
52. O'Neill J, Tabish H, Welch V, Petticrew M, Pottie K, Clarke M, et al. Applying an equity lens to interventions: using PROGRESS ensures consideration of socially stratifying factors to illuminate inequities in health. J Clin Epidemiol. 2014;67(1):56-64.
53. Welch V, Petticrew M, Petkovic J, Moher D, Waters E, White H, et al. Extending the PRISMA statement to equity-focused systematic reviews (PRISMA-E 2012): explanation and elaboration. Journal of Clinical Epidemiology. 2016;70:68-89.
54. Zorzela L, Loke YK, Ioannidis JP, Golder S, Santaguida P, Altman DG, et al. PRISMA harms checklist: improving harms reporting in systematic reviews. BMJ. 2016;352.
55. Noyes J, Booth A, Moore G, Flemming K, Tunçalp Ö, Shakibazadeh E. Synthesising quantitative and qualitative evidence to inform guidelines on complex interventions: clarifying the purposes, designs and outlining some methods. BMJ Global Health. 2019;4(Suppl 1):e000893.
56. Hong QN, Pluye P, Bujold M, Wassef M. Convergent and sequential synthesis designs: implications for conducting and reporting systematic reviews of qualitative and quantitative evidence. Syst Rev. 2017;6(1):61-.
57. Munabi-Babigumira S, Glenton C, Lewin S, Fretheim A, Nabudere H. Factors that influence the provision of intrapartum and postnatal care by skilled birth attendants in low-and middle-income countries: a qualitative evidence synthesis. Cochrane Database of Systematic Reviews. 2017(11).

For peer review only

58. Balshem H, Helfand M, Schunemann HJ, Oxman AD, Kunz R, Brozek J, et al. GRADE guidelines: 3. Rating the quality of evidence. J Clin Epidemiol. 2011;64(4):401-6.
59. Lewin S, Booth A, Glenton C, Munthe-Kaas H, Rashidian A, Wainwright M, et al. Applying GRADE-CERQual to qualitative evidence synthesis findings: introduction to the series. Implementation Science. 2018;13(1):2.
60. Rehfuess EA, Booth A, Brereton L, Burns J, Gerhardus A, Mozygemba K, et al. Towards a taxonomy of logic models in systematic reviews and health technology assessments: A priori, staged, and iterative approaches. Research synthesis methods. 2018;9(1):13-24.
61. Evans RE, Craig P, Hoddinott P, Littlecott H, Moore L, Murphy S, et al. When and how do ‘effective’ interventions need to be adapted and/or re-evaluated in new contexts? The need for guidance. Journal of Epidemiology and Community Health. 2019;73(6):481-2.
62. Craig P, Dieppe P, Macintyre S, Michie S, Nazareth I, Petticrew M, et al. Developing and evaluating complex interventions: the new Medical Research Council guidance. BMJ (Clinical research ed). 2008;337:a1655-a.

For peer review only

Authors' Contributions

All authors contributed to the conception of the protocol and the design of the systematic review. RE drafted the protocol. All authors read and approved the final manuscript. RE is the guarantor of the review.

Funding

This work was supported by the NIHR-PHR grant number NIHR129113.

Competing Interests

None declared.

For peer review only

Figure 1. CHIMES Review Results-based Convergent Synthesis Design

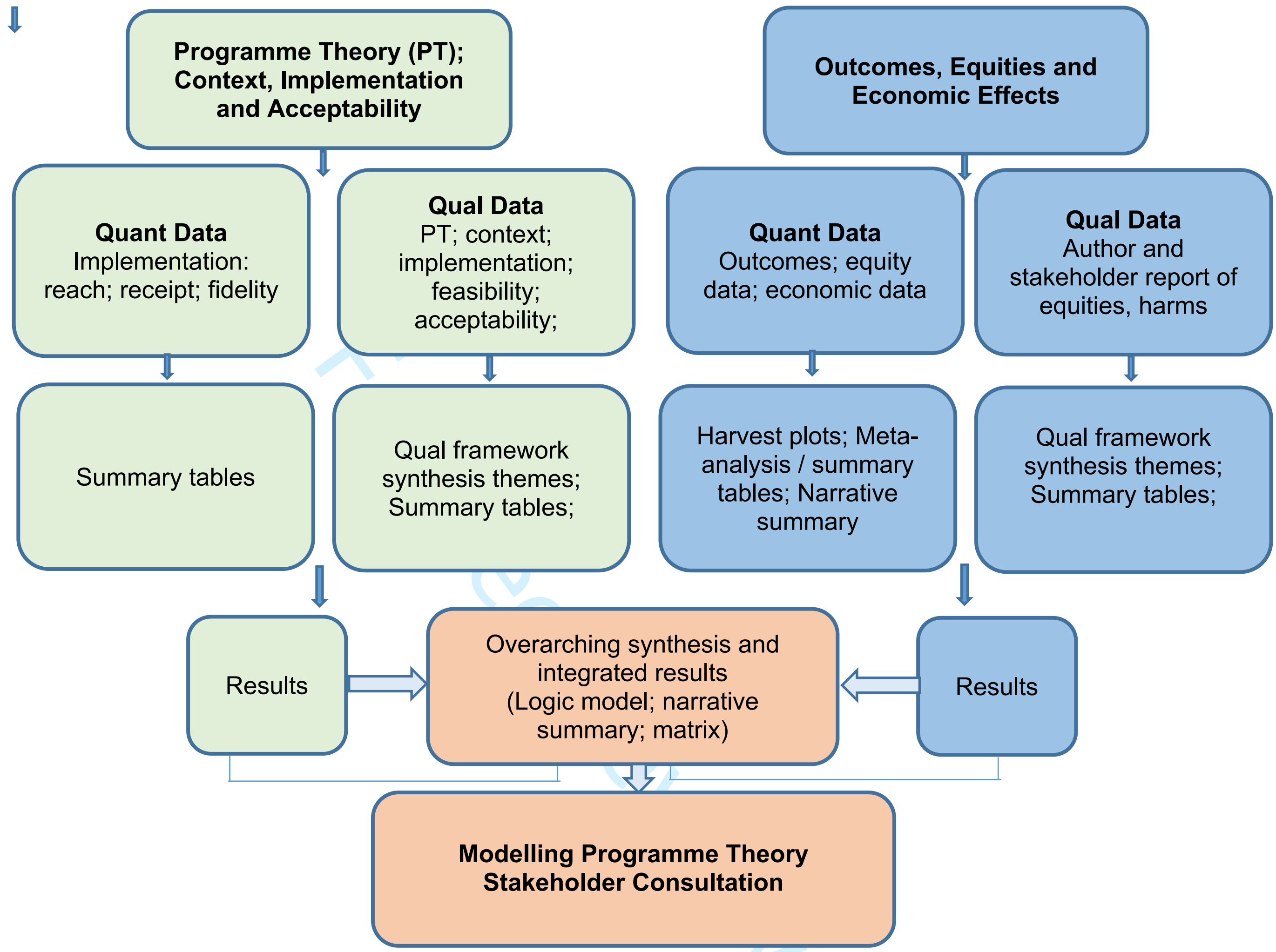

Figure 2. Intervention Prioritisation for Development, Adaptation and Evaluation in UK Context

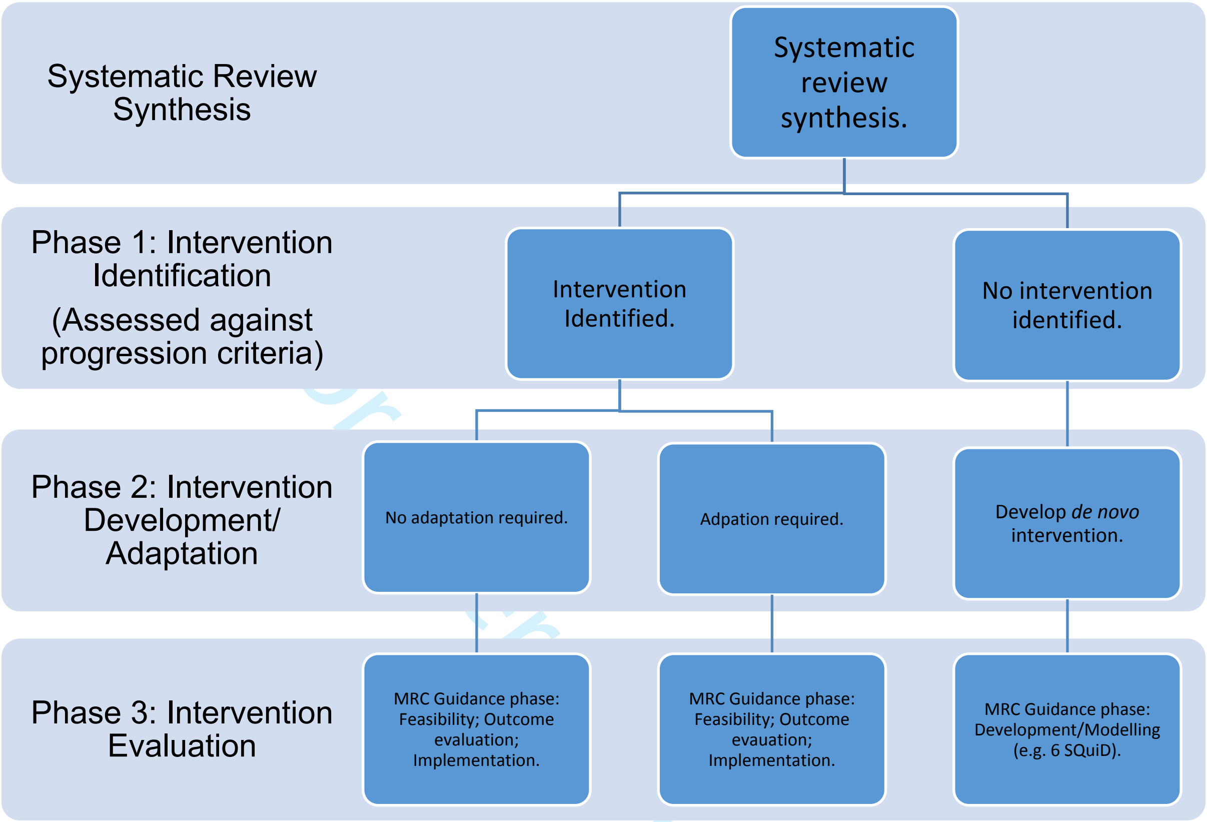

Supplement B: CHIMES Review Search Strategy (Developed in Ovid MEDLINE)

1 exp Child/
2 exp Infant/
3 Young Adult/
4 Adolescent/
5 (teen or teens or teenager*).tw.
6 (adolesc* or preadolesc* or pre-adolesc* or juvenile*).tw.
7 (youth or youths or youngster*).tw.
8 ((young adj (person or persons or people)) or "early adult*").tw.
9 (student or students or schoolchild*).tw.
10 (girl* or boy* or child or children or infant or infants or kid or kids).tw.
11 (pediatri* or paediatric*).tw.
12 (pubescen* or puberty or prepubescen* or pre-pubescen*).tw.
13 orphan*.tw.
14 Child, Foster/
15 Child, Orphaned/
16 "Child of Impaired Parents"/
17 1 or 2 or 3 or 4 or 5 or 6 or 7 or 8 or 9 or 10 or 11 or 12 or 13 or 14 or 15 or 16
18 ((substitute or "local authority" or state or statutory or public or "out of home" or order or place* or group*) adj (care or placement*)).tw.
19 ((nonparent or non-parent) adj3 care).tw.
20 ((children's or childrens) adj home).tw.
21 ((institution* or residential or foster or kinship or group) adj3 (care or home* or placement*)).tw.
22 ("support* living" or "supported lodging*" or "care leaver*").tw.
23 (leaving adj2 care).tw.
24 ((in or welfare or social or respite) adj care).tw.
25 looked after.tw.
26 Special guardian*.tw.
27 Foster Home Care/
28 Child, Institutionalized/
29 18 or 19 or 20 or 21 or 22 or 23 or 24 or 25 or 26 or 27 or 28
30 "Quality of Life"/
31 personal satisfaction/
32 (wellbeing or well-being or "well being").tw.
33 (illbeing or ill-being or "ill being").tw.
34 hedoni*.tw.
35 (eudaimoni* or eudaemoni* or eudemoni*).tw.
36 happiness.tw.
37 ((positive or negative) adj affect).tw.
38 flourish*.tw.
39 ("life satisfaction" or "satisfaction with life").tw.
40 ("positive and negative affect schedule" or PANAS).tw.
41 ("Warwick-Edinburgh Mental Wellbeing" or WEMWBS).tw.
42 ("State Trait Anxiety Inventory" or STAI).tw.
43 "Perceived Stress Scale".tw.
44 SWLS.tw.
45 30 or 31 or 32 or 33 or 34 or 35 or 36 or 37 or 38 or 39 or 40 or 41 or 42 or 43 or 44
46 Mental Health/
47 exp Mental Disorders/
48 Catatonia/
49 Self-Injurious Behavior/
50 Self Mutilation/

For peer review only

51 Suicide/
52 Suicidal Ideation/
53 Suicide, Attempted/
54 Suicide, Completed/
55 "mental health".tw.
56 ("bodily distress" or paraphilic or paraphilia or catatonia or catatonic or dissociation or "impulse control").tw.
57 (schizophrenia or psychotic or psychosis or OCD or "obsessive compulsive disorder").tw.
58 suicid*.tw.
59 (self adj2 (harm or injur* or cutting or mutilation or poison* or burn*)).tw.
60 (("post traumatic" or post-traumatic or posttraumatic) adj2 (stress or disorder*)).tw.
61 ((grief or adjustment or "reactive attachment" or "disinhibited social engagement") adj2 (disorder* or condition* or problem*)).tw.
62 (disruptive adj2 behavio?r*).tw.
63 ((behavio?r* or neurodevelopmental or mood or fear or anxiety or personality or disruptive or dissocial or impulse or factitious or neurocognitive or feeding or eating or elimination or disruptive or dissocial or anxiety or depressive) adj3 (disorder* or condition* or problem*)).tw.
64 46 or 47 or 48 or 49 or 50 or 51 or 52 or 53 or 54 or 55 or 56 or 57 or 58 or 59 or 60 or 61 or 62 or 63
65 45 or 64
66 exp Controlled Clinical Trial/
67 Double-Blind Method/
68 exp "Costs and Cost Analysis"/
69 random allocation/
70 (("pre-test" or pretest or posttest or "post-test" or "pre-intervention" or "post-intervention" or "controlled before" or "before and after" or "follow-up assessment") and (controlled or control or "comparison participants" or "comparison group" or "usual care" or placebo)).tw.
71 ("quasi-experiment*" or quasiexperiment* or "quasi-randomi*" or "quasirandomi*" or "natural* experiment" or "time series" or "interrupted time").tw.
72 ((controlled or control or intervention or comparison) adj3 (group or groups or study or trial or evaluation or cohort or cohorts or longitudinal or matched or matching or experiment or experimental)).tw.
73 ("difference in difference" or "instrumental variable*" or "propensity score matching" or "process evaluation").tw.
74 ((cost or costs or costing or economic) adj1 (analysis or effectiveness or benefit or evaluation or utility or savings or measure or measures)).tw.
75 (trial or "randomi?ed controlled trial" or rct or "cross-over design" or " cross over design" or "crossover design" or "cross-over study" or "cross over study" or "crossover study" or "factorial design" or "controlled study" or "controlled design" or "single-blind" or "single blind" or "double-blind" or "double blind" or "triple-blind" or "triple blind").tw.
76 effectiveness.tw.
77 program evaluation/
78 (program* adj (effect* or efficacy)).tw.
79 ((theor* or mechanism*) adj3 (change or impact or program*)).tw.
80 (("mixed method*" or "mixed-method*" or process or qualitative) adj3 evaluation*).tw.
81 (causal adj2 (assumption* or process*)).tw.
82 66 or 67 or 68 or 69 or 70 or 71 or 72 or 73 or 74 or 75 or 76 or 77 or 78 or 79 or 80 or 81
83 17 and 29 and 65 and 82

For peer review only

Supplement A: CHIMES Review Logic Model

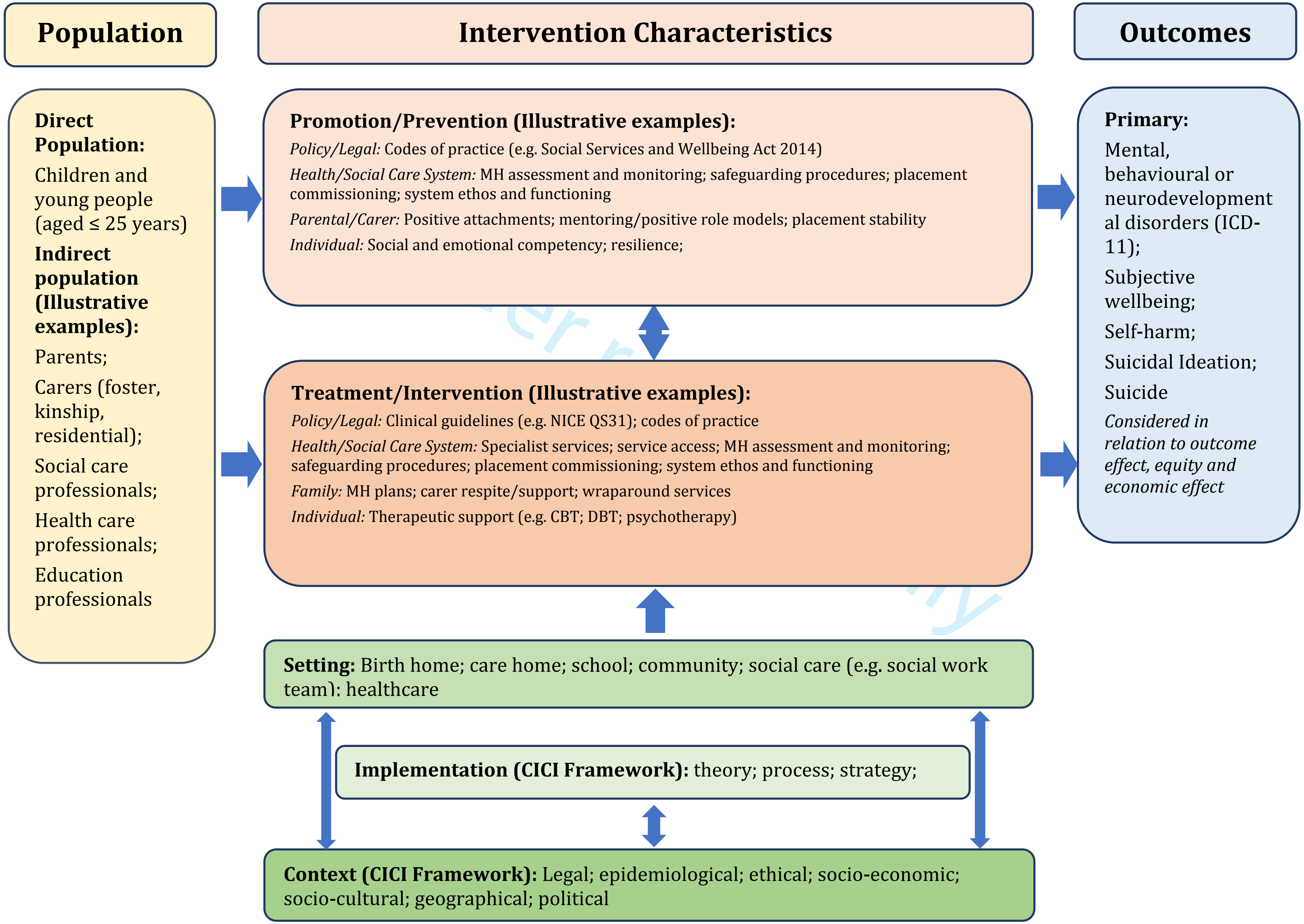

PRISMA-P (Preferred Reporting Items for Systematic review and Meta-Analysis Protocols) 2015 checklist: recommended items to address in a systematic review protocol*

Section and topic	Item No	Checklist item	Location in Text
**ADMINISTRATIVE INFORMATION**			
Title:			
Identification	1a	Identify the report as a protocol of a systematic review	p2
Update	1b	If the protocol is for an update of a previous systematic review, identify as such	N/A
Registration	2	If registered, provide the name of the registry (such as PROSPERO) and registration number	p2
Authors:			
Contact	3a	Provide name, institutional affiliation, e-mail address of all protocol authors; provide physical mailing address of corresponding author	p1
Contributions	3b	Describe contributions of protocol authors and identify the guarantor of the review	p19
Amendments	4	If the protocol represents an amendment of a previously completed or published protocol, identify as such and list changes; otherwise, state plan for documenting important protocol amendments	N/A
Support:			
Sources	5a	Indicate sources of financial or other support for the review	p19
Sponsor	5b	Provide name for the review funder and/or sponsor	p19
Role of sponsor or funder	5c	Describe roles of funder(s), sponsor(s), and/or institution(s), if any, in developing the protocol	p19
**INTRODUCTION**			
Rationale	6	Describe the rationale for the review in the context of what is already known	pp. 4-5
Objectives	7	Provide an explicit statement of the question(s) the review will address with reference to participants, interventions, comparators, and outcomes (PICO)	pp. 5-6
**METHODS**			
Eligibility criteria	8	Specify the study characteristics (such as PICO, study design, setting, time frame) and report characteristics (such as years considered, language, publication status) to be used as criteria for eligibility for the review	pp. 6-7
Information sources	9	Describe all intended information sources (such as electronic databases, contact with study authors, trial registers or other grey literature sources) with planned dates of coverage	p7
Search strategy	10	Present draft of search strategy to be used for at least one electronic database, including	p7 /Supplement B

For peer review only

		planned limits, such that it could be repeated	
Study records:			
Data management	11a	Describe the mechanism(s) that will be used to manage records and data throughout the review	p8
Selection process	11b	State the process that will be used for selecting studies (such as two independent reviewers) through each phase of the review (that is, screening, eligibility and inclusion in meta-analysis)	p8
Data collection process	11c	Describe planned method of extracting data from reports (such as piloting forms, done independently, in duplicate), any processes for obtaining and confirming data from investigators	p8
Data items	12	List and define all variables for which data will be sought (such as PICO items, funding sources), any pre-planned data assumptions and simplifications	pp.8-9
Outcomes and prioritization	13	List and define all outcomes for which data will be sought, including prioritization of main and additional outcomes, with rationale	pp.6-7/ Supplement A
Risk of bias in individual studies	14	Describe anticipated methods for assessing risk of bias of individual studies, including whether this will be done at the outcome or study level, or both; state how this information will be used in data synthesis	p9
Data synthesis	15a	Describe criteria under which study data will be quantitatively synthesised	pp.9-11
	15b	If data are appropriate for quantitative synthesis, describe planned summary measures, methods of handling data and methods of combining data from studies, including any planned exploration of consistency (such as I^2, Kendall's τ)	pp.9-11
	15c	Describe any proposed additional analyses (such as sensitivity or subgroup analyses, meta-regression)	pp.9-11
	15d	If quantitative synthesis is not appropriate, describe the type of summary planned	
Meta-bias(es)	16	Specify any planned assessment of meta-bias(es) (such as publication bias across studies, selective reporting within studies)	
Confidence in cumulative evidence	17	Describe how the strength of the body of evidence will be assessed (such as GRADE)	p11

*** It is strongly recommended that this checklist be read in conjunction with the PRISMA-P Explanation and Elaboration (cite when available) for important clarification on the items. Amendments to a review protocol should be tracked and dated.** **The copyright for PRISMA-P (including checklist) is held by the PRISMA-P Group and is distributed under a Creative Commons Attribution Licence 4.0.**

From: Shamseer L, Moher D, Clarke M, Ghersi D, Liberati A, Petticrew M, Shekelle P, Stewart L, PRISMA-P Group. Preferred reporting items for systematic review and meta-analysis protocols (PRISMA-P) 2015: elaboration and explanation. BMJ. 2015 Jan 2;349(jan02 1):g7647.

For peer review only

BMJ Open

Care-experienced cHildren and young people's Interventions to improve Mental health and wEllbeing outcomes: Systematic review (CHIMES) Protocol

Journal:	*BMJ Open*
Manuscript ID	bmjopen-2020-042815.R1
Article Type:	Protocol
Date Submitted by the Author:	14-Oct-2020

Complete List of Authors:	Evans, Rhiannon; Cardiff University, DECIPHer, School of Social Sciences<br>Boffey, Maria; The Fostering Network<br>MacDonald, Sarah; Cardiff University, DECIPHer, School of Social Sciences<br>Noyes, Jane; Bangor University, School of Social Sciences<br>Melendez-Torres, G.J; University of Exeter,<br>Morgan, Helen; Cardiff University, SURE, University Library Service<br>Trubey, Robert ; Cardiff University, Centre for Trials Research<br>Robling, Michael; Cardiff University, General Practice<br>Willis, Simone; Cardiff University, Specialist Unit for Review Evidence<br>Wooders, Charlotte; The Fostering Network

**Primary Subject Heading**:	Mental health
Secondary Subject Heading:	Evidence based practice, Health economics, Mental health, Public health, Research methods
Keywords:	PUBLIC HEALTH, MENTAL HEALTH, Child & adolescent psychiatry < PSYCHIATRY, Child protection < PAEDIATRICS

SCHOLARONE™ Manuscripts

I, the Submitting Author has the right to grant and does grant on behalf of all authors of the Work (as defined in the below author licence), an exclusive licence and/or a non-exclusive licence for contributions from authors who are: i) UK Crown employees; ii) where BMJ has agreed a CC-BY licence shall apply, and/or iii) in accordance with the terms applicable for US Federal Government officers or employees acting as part of their official duties; on a worldwide, perpetual, irrevocable, royalty-free basis to BMJ Publishing Group Ltd ("BMJ") its licensees and where the relevant Journal is co-owned by BMJ to the co-owners of the Journal, to publish the Work in this journal and any other BMJ products and to exploit all rights, as set out in our licence.

The Submitting Author accepts and understands that any supply made under these terms is made by BMJ to the Submitting Author unless you are acting as an employee on behalf of your employer or a postgraduate student of an affiliated institution which is paying any applicable article publishing charge ("APC") for Open Access articles. Where the Submitting Author wishes to make the Work available on an Open Access basis (and intends to pay the relevant APC), the terms of reuse of such Open Access shall be governed by a Creative Commons licence – details of these licences and which Creative Commons licence will apply to this Work are set out in our licence referred to above.

Other than as permitted in any relevant BMJ Author's Self Archiving Policies, I confirm this Work has not been accepted for publication elsewhere, is not being considered for publication elsewhere and does not duplicate material already published. I confirm all authors consent to publication of this Work and authorise the granting of this licence.

For peer review only

Care-experienced cHildren and young people's Interventions to improve Mental health and wEllbeing outcomes: Systematic review (CHIMES) Protocol

Dr Rhiannon Evans[1]
Corresponding Author
Email: EvansRE8@cardiff.ac.uk
Tel: 02920 870099

Maria Boffey[2] Email: Maria.Boffey@fostering.net

Dr Sarah MacDonald[1] Email: MacDonaldS@cardiff.ac.uk

Prof Jane Noyes[3] Email: jane.noyes@bangor.ac.uk

Prof GJ Melendez Torres[4] Email: g.j.melendez-torres@exeter.ac.uk

Dr Helen Morgan[5] Email: MorganHE1@cardiff.ac.uk

Dr Rob Trubey[6] Email: TrubeyRJ@cardiff.ac.uk

Prof Michael Robling[6] Email: RoblingMR@cardiff.ac.uk

Simone Willis[5] Email: WillisS5@cardiff.ac.uk

Charlotte Wooders[2] Email: Charlotte.Wooders@fostering.net

[1]DECIPHer, School of Social Sciences, Cardiff University, 1-3 Museum Place, Cardiff, CF10 3BD
[2]The Fostering Network, 33 Cathedral Road, Cardiff, CF11 9HB
[3] School of Health Sciences, Bangor University, Bangor, LL57 2DG
[4]Peninsula Technology Assessment Group (PenTAG), University of Exeter, South Cloisters, St Luke's Campus, Exeter, EX1 2LU.
[5]Specialist Unit for Review Evidence, Cardiff University, Neuadd Meirionnydd, Heath Park Campus, Cardiff, CF14 4YS
[6] Centre for Trials Research, Cardiff University, Neuadd Meirionnydd, Heath Park Campus, Cardiff, CF14 4YS

For peer review only

Abstract

Introduction: The mental health and wellbeing of children and young people who have been in care (i.e. care-experienced) is a priority. There are a range of interventions aimed at addressing these outcomes, but the international evidence-base remains ambiguous. There is a paucity of methodologically robust systematic reviews of intervention effectiveness, with few considering the contextual conditions under which evaluations were conducted. This is important in understanding the potential transferability of the evidence-base across contexts. The present systematic review will adopt a complex systems perspective to synthesise evidence reporting evaluations of mental health and wellbeing interventions for care-experienced children and young people. It will address impact, equity, cost-effectiveness, context, implementation and acceptability. Stakeholder consultation will prioritise a programme theory, and associated intervention, that may progress to further development and evaluation in the UK.

Methods and Analysis: We will search 15 bibliographic databases from 1990-June 2020. Supplementary searching will include citation tracking, author recommendation, and identification of evidence clusters relevant to included evaluations. The eligible population is children and young people (aged ≤ 25 years) with experience of being in care. Outcomes are 1) mental, behavioural or neurodevelopmental disorders; 2) subjective wellbeing; 3) self-harm; suicidal ideation; suicide. Study quality will be appraised with methodologically appropriate tools. We will construct a taxonomy of programme theories and intervention types. Thematic synthesis will be used for qualitative data reporting context, implementation and acceptability. If appropriate, meta-analysis will be conducted with outcome and economic data. Convergent syntheses will be used to integrate syntheses of qualitative and quantitative data.

Ethics and Dissemination: We have a comprehensive strategy for engagement with care-experienced children and young people, carers and social care professionals. Dissemination will include academic and non-academic publications and conference presentations. Ethical approval from Cardiff University's School of Social Sciences REC will be obtained if necessary.

PROSPERO Registration: CRD42020177478

For peer review only

Strengths and limitations of this study

- This review will offer one of the first methodologically robust syntheses of evidence reporting evaluations of mental health and wellbeing interventions for care-experienced children and young people;
- In taking a complex systems approach, the review will provide insights into the potential transferability of interventions and the replicability of effects across contexts;
- From this review, the study team aim to identify a potential programme theory (and associated intervention) that can progress to further development and evaluation in the UK context;
- The review will support policymakers and practitioners in decision-making about how to support the mental health and wellbeing of children and young people who have been in care;
- The review is limited by only including studies published in higher resource countries.

For peer review only

Background

Rationale

The mental health and wellbeing of children and young people who have been in care (i.e. care-experienced) is a growing public health and social care concern. Internationally, almost 50% of individuals involved in the child welfare system have a diagnosable mental health condition (1). Meanwhile in the UK, they are nearly five times as likely to have at least one psychiatric diagnosis compared to the general population (2). Care-experienced individuals are at an elevated risk of poor subjective wellbeing (3) and are more than four times as likely to attempt suicide (3). Adverse mental health outcomes incur significant health and social care costs, often due to the associated risk of placement instability and breakdown (4, 5). Evidence from the UK reports that insecure and unstable care placements cost £22,415 more per child per annum (across health, social care and criminal justice) than stable care pathways (6).

There is a clear need to improve mental health provision for those who experience care, with NICE guidance stating that the UK evidence base does not adequately serve this population (7). Equally, while the Department for Education and Department of Health and Social Care's joint statutory guidance has mandated that local authorities ensure timely and adequate services (8), there are reported incidents of failure to identify need, overly stringent eligibility thresholds, and withholding of care where there is not a stable placement (9). A comprehensive synthesis is required to identify evidence-based interventions in order to develop recommendations to enhance policy and practice.

Internationally there is a large evidence base for interventions (10-21), although in the UK it remains emergent. Current interventions focus on preventative approaches that operate across socio-ecological domains, including community level change (e.g. Flying Start) (22), functioning within the care placement (23, 24), the theoretical orientation of social care teams (e.g. adoption of a trauma informed model) (25), and the social and emotional competencies of the individual child or young person (26). Meanwhile, treatment has tended to focus on both the type and availability of therapeutic approaches, such as counselling or access to mental health services (27).

Despite the size of the extant literature, there are limitations that need to be attended to, notably in regard to existing systematic reviews. First, the most inclusive syntheses lack rigorous methodologies. One of the most recent and relevant literature reviews recognised that it is not exhaustive (10). Second, reviews tend to focus on a limited number of intervention types (e.g. Treatment Foster Care) (11), populations (e.g. foster care) (11, 13, 17, 26, 27), or outcomes (e.g. externalising behaviours) (17, 18). This makes it difficult to know if a particular

For peer review only

intervention can work effectively with different populations across multiple outcomes in order to reduce the number of approaches needing to be resourced within a system. Third, reviews often include only rudimentary treatment of context and process data. Understanding the complex system in which interventions operate is imperative. International variations in social care and health care systems may constrain the transferability of evidence and the extent to which we can 'borrow strength' from the original evaluation (28). For example, Multi-systematic therapy (MST) is shown to not be effective in Sweden, despite demonstrating effects in the USA, as it is essentially equal to usual care (29).

The present mixed method systematic review will use a robust methodology to draw together the evidence-base for interventions aimed at improving the mental health and wellbeing of care-experienced children and young people, including those currently in care and those who have previously been in care. Adopting a complex systems perspective (30, 31), we will synthesise evidence of impact, equity and cost effectiveness, while also focusing on understanding how intervention effects are contingent on the context in which they are evaluated. In collaboration with stakeholders (young people, carers and social care professionals), the resulting synthesis will be used to identify, where possible, an overarching programme theory (or theories) that may inform the development or adaptation of an intervention to progress to further evaluation within the UK context.

Review Aims and Questions

The study aims to systematically review and synthesise international evidence on interventions to improve the mental health and wellbeing of care-experienced children and young people. We will address the following research questions:

RQ1: What are the types, theories and outcomes tested in mental health and wellbeing interventions for care-experienced children and young people?

RQ2: What are the effects (including inequities and harms) and economic effects of interventions?

RQ3: How do contextual characteristics shape implementation factors, and what are key enablers and inhibitors of implementation?

RQ4: What is the acceptability of interventions to key stakeholders?

RQ5: Can and how might intervention types, theories, components, and outcomes be related in an overarching system-based programme theory?

On the completion of the initial phases of the systematic review (RQ1-5), the study will undertake a second phase of research and address a further research question:

For peer review only

RQ6: Drawing on the findings from RQ1-5, what do stakeholders think is the most effective, feasible and acceptable intervention in the UK that could progress to further outcome or implementation evaluation?

Methods

Protocol and Registration

The protocol is registered with PROSPERO and reported in accordance with the Preferred Reporting Items for Systematic reviews and Meta-Analyses Protocols (PRISMA-P) guidelines (32). The review will be published in accord with the Preferred Reporting Items for Systematic reviews and Meta-Analyses (33), with qualitative synthesis being structured by the ENTREQ reporting standards (34) and the mixed methods process evaluation synthesis following the principles prescribed by Cochrane (35).

Eligibility Criteria

The eligibility parameters are reported in accordance with the PICOS framework and presented in the logic model (Supplement A).

Population: Children and young people aged $\leq$ 25 years olds. Where the target population extends beyond the age of 25 years old (e.g. 18-30 years old), subgroup data must be provided for participants aged $\leq$ 25 years olds. Currently placed in care or previous care experience. Period of time in care is not restricted. Care can include in-home care (i.e. voluntary transfer of parental responsibility to statutory services) and out-of-home care (e.g. foster care, residential care, or formal kinship care). No restriction on baseline mental health conditions or uptake of pharmacological treatment. The following are excluded: general population; children in need; individuals at the edge of care; care without specified statutory involvement (e.g. informal kinship care); adoption; and refugees. Any population may be targeted as intervention participants (e.g. carer, birth family, teacher or social worker) but outcomes must be reported at the level of the child or young person.

Intervention: Any attempt to disrupt existing system practices. They may be mono-component or multi-component and operate across any of the following socio-ecological domains: individual; inter-personal; organisational; community; and policy/legal. Interventions may focus on promotion/prevention or the management/treatment of symptomology. There are no a-priori criteria for implementation (i.e. delivery setting, delivery mode, delivery agent). The following are excluded: pharmacological interventions, either as a single component approach or multi-component intervention (e.g. combined with psychosocial activities).

Comparator: Treatment as usual; other active treatment; no specified treatment.

For peer review only

Outcomes: Three domains of primary outcomes: 1) Mental, behavioural or neurodevelopmental disorders as specified by ICD 11; 2) Subjective wellbeing (eudaimonia and hedonia); 3) Self-harm; suicidal ideation; suicide. Measures may use dichotomous, categorical or continuous variables. Outcomes may be ascertained through clinical assessment, self-report or report by another informant (e.g. teacher).

Study Design: Programme theory: Describe intended theory, logic model or mechanisms of effect. May include mediation analysis (RQ1); *Outcome evaluation:* (Individual/cluster) randomised controlled trials and quasi-experimental study designs. We exclude post measurement only or pre-post measurement in intervention group only (RQ1; RQ2); *Process evaluation:* All qualitative and quantitative study designs (RQ1; RQ3; RQ4). Included studies must empirically report on relevant contextual influences, implementation or acceptability; *Economic evaluation*: Must relate costs to benefits and can report: cost-minimisation, cost-effectiveness, cost utility or cost-benefit analysis. May be model-based or trial-based. Decision-analytic models capturing intervention impacts on mental health and wellbeing will be eligible (RQ2). *Relationship Between Study Designs:* To be included programme theory papers must have an associated empirical outcome evaluation. Process evaluations and economic evaluations do not necessarily have to be linked to an empirical outcome evaluation, as they provide wider contextual insight into how interventions interact with complex system characteristics.

Information Sources

The following electronic bibliographic databases will be searched: ASSIA, British Education Index, Child Development & Adolescent Studies, CINAHL, Embase, ERIC, Cochrane Database of Clinical Controlled Trials (CENTRAL), HMIC, IBSS, Medline (Medline in Process and Medline ePub), PsycINFO, Scopus, Social Policy & Practice, Social Services Abstracts, Sociological Abstracts and Web of Science (Social Sciences Citation Index, Conference Proceedings Citation Index- Social Science & Humanities, Emerging Sources Citation Index). Supplementary searching techniques will include citation tracking of included studies, contacting international experts, searching trial registers and consulting websites of key social and health care organisations. Searches will be conducted for 'clusters' of reports related to included studies to develop descriptions of programme theories and further progress understanding of context (36, 37). Inclusion will be restricted from 1990, which marked the ratification of the UN Convention on the Rights of the Child (38). This prescribed comprehensive social and health care provision for children internationally and started the proliferation of intervention in this area. To maximise applicability of evidence to the UK, studies from low- and middle- income countries will be excluded. All languages will be included in searches.

Search Strategy

For peer review only

We developed a provisional search strategy in Ovid MEDLINE (Supplement B). It will be adapted to the functionality of each database on study commencement.

Data Management and Study Selection

Data will be exported to Endnote for de-duplication and then to EPPI Reviewer Web for screening. In the first instance reference titles will be screened on the basis of title to identity clearly irrelevant retrievals (e.g. pharmacological treatments), with these exclusions being verified by a second reviewer. Titles and abstracts of remaining studies will be screened independently and in duplicate. The inclusion criteria pro-forma will be piloted and calibrated by two reviewers screening the same 100 title and abstracts, with disagreement being resolved through consensus or recourse to a third reviewer. Full texts will be retrieved and appraised for study inclusion. Conflicts will be resolved through discussion or recourse to a third reviewer. A record of the selection process will be retained in adherence with the PRISMA flow diagram.

Data Collection

Once the final number of included studies is confirmed, we will chart clusters of studies, constructing a visual knowledge map similar to that recommended within scoping reviews (39, 40). This process will support decision-making about the depth of data extraction at the next stage (e.g. sampling may be conducted for qualitative studies) and extent of synthesis to be undertaken (e.g. meta-analysis may be conducted where there are multiple studies). The CHIMES Review has a Study Advisory Group that will be consulted at this stage to confirm the extraction and synthesis.

Standardised data extraction forms will be developed and calibrated with a subset of studies for input to EPPI Reviewer Web. Two versions of extraction forms may be developed based on the mapping of the data. The first will be for studies included in the review but not included in more in-depth synthesis, and the second for studies subjected to more in-depth synthesis. For primary qualitative studies a subset of data will be coded in vivo to develop a preliminary coding tree with a priori codes, but which can be amended to incorporate emergent codes. On confirmation of the extraction forms and coding tree, two reviewers will independently extract and code data from 10% of studies, with the remainder being extracted by one reviewer and verified by a second.

Data Items

For all studies data will be extracted on: study characteristics, participant demographics; setting; study design and methods; intervention theory of change and intervention characteristics. For outcome evaluations data will be extracted for: measurements, sequence generation, allocation concealment, blinding, data completeness, baseline differences and

For peer review only

adjustment for difference, control of confounding, and outcomes at follow-up at both population and sub-group level. Data items from process evaluations will be the implementation theory, implementation strategy and implementation agents. Quantifiable assessments of implementation will be reach, receipt, and fidelity. Contextual characteristics will be classified according to the CICI framework: geographical; epidemiological; legal; socio-economic; socio-cultural; ethical; and political (41). Acceptability data across stakeholders will be extracted. Economic evaluation data items will be intervention and comparator costs, perspective, structural and empirical inputs, time horizon, and cost-effectiveness. Health utility data will also be extracted. Where there is incomplete data the author will be asked for additional information and where this is not retrieved the data will be reported as missing and included in the risk of bias assessment.

Risk of Bias (Individual Studies)

Study data will be appraised with a methodologically appropriate tool. Programme theory will be appraised using a tailored appraisal tool developed by members of the research team (42). Domains of assessment will be: clarity of constructs; clarity of relationships between constructs; testability; parsimony and generalisability.

Outcome evaluations will be assessed with the relevant tool prescribed by the Cochrane Handbook for Systematic Reviews of Interventions (43). In the case of randomised controlled trials, risk of bias will be identified across seven domains: sequence generation; allocation concealment; blinding of participants, personnel and outcome assessments; completeness of outcome data; selective reporting of outcomes; other sources of bias. Each domain will be rated as high risk, low risk or unclear risk.

Qualitative data within process evaluations will be appraised using a tool developed in previous systematic reviews (44, 45). It will address two key domains: reliability and trustworthiness (sampling rigour; data collection rigour; analysis rigour; data supporting analysis); and usefulness (breadth/depth of findings; privileges participant perspectives). Two global assessments of overall reliability/trustworthiness and overall usefulness will then be made. Domains are rated as high, medium or low.

Economic data will be appraised with the Drummond checklist (46). It will assess if there are appropriate descriptions of comparators; identification and valuation of costs and consequences; discounting; and analysis of uncertainty. We will also consider the governance and ethical conduct of studies (47). All quality appraisal will be undertaken independently and in duplicate, with disagreement being resolved through discussion or recourse to a third reviewer.

For peer review only

Synthesis of Results

Programme Theory, Context, Implementation and Acceptability (RQ1; RQ4; RQ5). We will construct a single taxonomy describing intervention types, theories and outcomes. It will be used to understand if there are different or dominant theories according to different types of interventions and/or outcomes (42). Context and implementation will be classified according to the CICI framework (41). Implementation will also comprise data on quantifiable assessments of implementation, including: reach; receipt; and fidelity. Acceptability will draw upon the understanding, as outlined by Medical Research Council guidance on process evaluations (48). It will include simplistic measures of satisfaction to more complex, often qualitative considerations of the experience of engaging with the intervention. Acceptability will be explored for participants, delivery agents and service funders.

Analysis will adhere to the five phases of framework synthesis (49-52): 1) Familiarisation with included studies; 2) Develop a thematic framework based on the parameters of the review. This will be refined and calibrated with a subset of data; 3) Index the remaining corpus of data. New themes, and associated codes, may be generated in vivo through the process of constant comparison across studies and against the framework; 4) Charting the data into a framework matrix summarizing data by category from each transcript.; 5) Mapping and interpreting data to create the typology of interventions and to explain associations between themes to help understand how and why interventions may or may not be effective. The synthesis may be presented graphically and narratively.

Intervention Outcome Effects, Equities and Economic Effects (RQ2). The taxonomy describing intervention types, theories and outcomes will inform and structure the analyses undertaken as part of the quantitative synthesis. Depending on intervention heterogeneity we will consider meta-analysing effect estimates from outcome evaluations. In preparation for meta-analysis, we will extract effect estimates from studies that will be classified into outcome domains. Where appropriate outcomes will be converted to odds ratios using logistic transformation for pooling. Estimates from cluster randomised trials will be checked for unit of analysis issues, and where necessary, an inflation factor will be applied to the standard error of effect estimates. Where intra-cluster correlation coefficients are not available and effect estimates have not been adjusted for clustering, we will impute an ICC using the average of estimates for specific outcomes from 'most similar' intervention evaluations.

We will undertake robust variance estimation meta-analyses according to intervention type, outcome and timepoint, considering up to six months from baseline as short-term, six months to two years as mid-term, and beyond two years as long-term. Robust variance estimation meta-analysis is a method that permits the inclusion of more than one effect estimate per study in a

For peer review only - http://bmjopen.bmj.com/site/about/guidelines.xhtml

meta-analysis; this is in contrast to standard meta-analysis models that assume independence between individual effect estimates. It is common in meta-analysis of psychosocial interventions for outcome evaluations to present multiple relevant effect estimates per outcome (e.g. multiple estimates of child behavioural problems). This method will permit use of all relevant information from included studies. Within each meta-analysis, we will examine heterogeneity using a combination of Cochran's Q, tau-squared and I^2. Where heterogeneity is substantial (I^2 >50%), we will scrutinise included studies to hypothesise and explore the reasons for this.

Equity effects will be categorized according to PROGRESS-Plus (53). Data will be reported in adherence to the PRISMA E-2012 extension (54). We will use harvest plots to assess equity effects and meta-regression to test whether characteristics of study populations are associated with effectiveness. Intervention harms will be treated in accordance with the PRISMA harms extension (55).

Economic evaluations will be summarized. Summarized data will include measures of costs, cost -effectiveness, indirect resource use, and whether a trial-based or model-based analysis was conducted. If there is sufficient homogeneity in measures across studies, these will be synthesised via meta-analysis. Measures of costs, cost-effectiveness and indirect resource use will be adjusted in line with inflation and currency to provide a contextually relevant estimate of costs in the current UK context.

Synthesis of qualitative and quantitative syntheses: The review will adhere to a convergent synthesis design (56, 57). This will entail the separate synthesis and reporting of qualitative and quantitative data in methodologically relevant but complementary manner, before integrating them in a further synthesis (Figure 1). At this stage the synthesis of context, implementation and acceptability will be integrated with outcome data to explain intervention effectiveness and potential inequities. To support this we will use a narrative summary and a 2x2 matrix that will map context, implementation, acceptability, participant values and costs against the included intervention theories/types (58). The matrix can demonstrate where an intervention may have (in)sufficiently accommodated factors that led to its (in)effectiveness, and what factors need to be better attended to moving forward. We will additionally develop a logic model(s) of a potentially effective programme theory or theories that is a best fit with the evidence base.

To support harmonisation and integration of syntheses across the review, we have formulated research questions that are complementary and contingent, where achieving a comprehensive answer to one question is dependent on the answers to other questions. When conducting the review, we will: 1) Have the same members of the research team work on both the synthesis of qualitative and quantitative data; 2) Screen all study types simultaneously and by the same members of the research team, storing data in EPPI Reviewer to ensure easy sharing of study

For peer review only

data across syntheses; and 3) Use method specific appraisal tools that have been combined in previous reviews due to providing epistemological flexibility or consonance. We will consider appraising the certainty of evidence generated through the different reviews with the compatible tools of GRADE (59) and GRADE CERQual (60).

Programme Theory Modelling, Stakeholder Consultation and Intervention Prioritisation

From the review findings, we will aim to identify an overarching programme theory (or compatible programme theories across socio-ecological domains), and associated interventions, which can address multiple outcomes across a number of populations (RQ5). These will be diagrammatically depicted with the use of system-based logic models and an accompanying narrative summary (61). Using the review logic model as a departure point, we will integrate review data to clarify and expand upon key intervention domains: theory (theories of change); setting; population; context; implementation; and outcomes. The logic mode (s)l then will illustrate the relationship between the underpinning theories of change, intervention effectiveness and important modifying factors that impact upon implementation and acceptability.

Stakeholder consultations will be held to prioritise the theories and interventions that might be most feasible and acceptable within the UK context and which may progress to further evaluation (RQ6) (See PPI section for stakeholder description). There are three phases of assessment as part of this prioritisation process (Figure 2):

Phase 1 Intervention Identification: Stakeholders will assess and rank candidate programme theories, and associated interventions, against the following progression criteria: 1) acceptability; 2) feasibility (particularly in UK context); and 3) perceived effectiveness. At the end of this phase there should be a potential programme theory and possible intervention components. Where no candidate theories meet the progression criteria, or an overarching theory is identified but there are no intervention components (e.g. no intervention embodies all of the interacting aspects of the theory), we will consider that de novo intervention development is required.

Phase 2 Intervention Development and Adaptation: If a programme theory is identified, and has an associated intervention, stakeholders will assess if adaptation is required for the UK context (if evaluated elsewhere). This will be done by mapping similarities in contexts using a simplified version of the CICI framework (41). If contexts are considered dissimilar, stakeholders will consider the types of adaptations that may be required. In progress MRC-NIHR funded guidance on adaptation will support this discussion (62). If a de novo intervention

For peer review only

is deemed necessary, we will ask stakeholders to undertake preliminary consultation on what an intervention might look like, considering what contextual factors need to be accommodated in order to make it acceptable, feasible and effective.

Phase 3 Intervention Evaluation: This phase will consider the appropriate research design for progressing the intervention. We will use the Medical Research Council's guidance on developing and evaluating complex interventions to define the phase of evaluation (63). To support decision making in the case where it is appropriate to evaluate an existing intervention (adapted or non-adapted), we will use the 'borrowing strength' framework to assess if any outcome data from the original context is applicable within the UK context (28). The framework dictates that if contexts are largely congruent or adaptations are minimal, then an implementation study may be warranted. Where contexts are significantly dissimilar or substantial adaptation has been undertaken, more extensive feasibility and outcome evaluation will be required. It is likely that decision-making at this phase will be predominantly conducted by the research team. On completion of the three prioritisation phases, we anticipate having a clearly identified research agenda to progress beyond the review.

Ethics and Dissemination

Ethics

Cardiff University's School of Social Sciences Research Ethics Committee will provide ethical approval for the stakeholder consultation if required.

Patient and Public Involvement

The review includes a comprehensive programme of patient and public (PPI) involvement. Engagement with children and young people will be facilitated through CASCADE Voices, a collaboration between Voices from Care Cymru and the Children's Social Care Research and Development Centre (CASCADE) at Cardiff University. CASCADE Voices comprises a research advisory group of care-experienced young people. We will consult with them at study commencement to refine and confirm the scope and focus of the review, notably the key subgroups to be analysed and the contextual influences on implementation.

To support the prioritisation of interventions moving forward, we will hold a consultation with CASCADE Voices. A social care practitioner workshop will be hosted with the ExChange Wales Network. The network, hosted by CASCADE, brings together social care sector stakeholders to share experiences and expertise. Further consultation opportunities will be sought with the UKRI funded Transdisciplinary Research for the Improvement of Young Mental Public Health (TRIUMPH) Network. THE CHIMES study Principal Investigator (RE) co-leads the Networks' programme on the mental health of key groups, which includes care-experienced young people.

For peer review only

Dissemination

Outputs from the study will be 1) NIHR PHR monograph; 2) academic publications reporting the results; and 3) a briefing report for policy and practitioners. Presentations will be delivered at academic, policy and practice workshops and conferences, both nationally and internationally. Networks and research infrastructures will be utilised to disseminate findings, notably TRIUMPH and the What Works Centre for Children's Social Care. The latter organisation was funded by the Department for Education and established in collaboration with Cardiff University, with the aim of improving outcomes for children in care. It has a focus on evidence synthesis, providing a depository of evidence summaries on their website for social care professionals. Further dissemination activities will be identified in collaboration with PPI stakeholders throughout the course of the study. Moving forward, sharing of knowledge on which interventions are likely to work means that finite public resources can be expended on approaches that can best ensure the positive mental health and wellbeing of care-experienced children and young people, while encouraging disinvestment from ineffective and potentially harmful approaches.

For peer review only

Figures

Figure 1: CHIMES Review Results-based Convergent Synthesis Design

Figure 2: Intervention Prioritisation for Development, Adaptation and Evaluation in UK Context

For peer review only

References

1. Bronsard G, Alessandrini M, Fond G, Loundou A, Auquier P, Tordjman S, et al. The Prevalence of Mental Disorders Among Children and Adolescents in the Child Welfare System: A Systematic Review and Meta-Analysis. Medicine. 2016;95(7):e2622-e.
2. Ford T, Vostanis P, Meltzer H, Goodman R. Psychiatric disorder among British children looked after by local authorities: Comparison with children living in private households. British Journal of Psychiatry. 2018;190(4):319-25.
3. Long SJ, Evans RE, Fletcher A, Hewitt G, Murphy S, Young H, et al. Comparison of substance use, subjective well-being and interpersonal relationships among young people in foster care and private households: a cross sectional analysis of the School Health Research Network survey in Wales. BMJ Open. 2017;7(2).
4. James S. Why Do Foster Care Placements Disrupt? An Investigation of Reasons for Placement Change in Foster Care. Social Service Review. 2004;78(4):601-27.
5. Rubin DM, Alessandrini EA, Feudtner C, Mandell DS, Localio AR, Hadley T. Placement Stability and Mental Health Costs for Children in Foster Care. Pediatrics. 2004;113(5):1336-41.
6. Curtis L, Burns A. Unit Costs of Health and Social Care 2017. Canterbury: Personal Social Services Research Unit, University of Kent; 2017.
7. NICE. Looked-after children and young people. Public health guideline [PH28]. London: National Institute for Health and Care Excellence; 2010.
8. Department for Education. Promoting the health and well-being of looked-after children. Statutory guidance for local authorities, clinical commissioning groups and NHS England. London: Department for Education; 2015.
9. House of Commons Education Committee. Mental health and well-being of looked-after children. Fourth Report of Session 2015–16. London: House of Commons Education Committee; 2016.
10. Luke N, Sinclair I, Woolgar M, Sebba J. What works in preventing and treating poor mental health in looked after children. London: NSPCC. 2014.
11. Turner W, Macdonald G. Treatment foster care for improving outcomes in children and young people: A systematic review. Research on Social Work Practice. 2011;21(5):501-27.
12. Everson-Hock ES, Jones R, Guillaume L, Clapton J, Goyder E, Chilcott J, et al. The effectiveness of training and support for carers and other professionals on the physical and emotional health and well-being of looked-after children and young people: a systematic review. Child Care Health Dev. 2012;38(2):162-74.
13. Hambrick EP, Oppenheim-Weller S, N'Zi A M, Taussig HN. Mental Health Interventions for Children in Foster Care: A Systematic Review. Child Youth Serv Rev. 2016;70:65-77.
14. Everson-Hock ES, Jones R, Guillaume L, Clapton J, Duenas A, Goyder E, et al. Supporting the transition of looked-after young people to independent living: a systematic review of interventions and adult outcomes. Child Care Health Dev. 2011;37(6):767-79.
15. Kerr L, Cossar J. Attachment Interventions with Foster and Adoptive Parents: A Systematic Review. Child Abuse Review. 2014;23(6):426-39.
16. O'Higgins A, Ott EM, Shea MW. What is the Impact of Placement Type on Educational and Health Outcomes of Unaccompanied Refugee Minors? A Systematic Review of the Evidence. Clinical child and family psychology review. 2018;21(3):354-65.
17. Marsh D. A systematic review and meta-analysis of randomized clinical trials of evidence-based practices through measured change of behavior for children in foster care 2017.
18. Solomon DT, Niec LN, Schoonover CE. The Impact of Foster Parent Training on Parenting Skills and Child Disruptive Behavior. Child Maltreat. 2017;22(1):3-13.
19. Sullivan AL, Simonson GR. A systematic review of school-based social-emotional interventions for refugee and war-traumatized youth. Review of Educational Research. 2016;86(2):503-30.

20. Macdonald GM, Turner W. Treatment foster care for improving outcomes in children and young people. The Cochrane database of systematic reviews. 2008(1):Cd005649.
21. Dickson K, Sutcliffe K, Gough D, Statham J. Improving the emotional and behavioural health of looked-after children and young people (LACYP): research review 2. London: Centre for Excellence and Outcomes in Children and Young People's Services (C4EO); 2009.
22. Hutchings J, Griffith N, Bywater T, Williams ME. Evaluating the Incredible Years Toddler Parenting Programme with parents of toddlers in disadvantaged (Flying Start) areas of Wales. Child: Care, Health and Development. 2017;43(1):104-13.
23. Sinclair I, Parry E, Biehal N, Fresen J, Kay C, Scott S, et al. Multi-dimensional Treatment Foster Care in England: differential effects by level of initial antisocial behaviour. European Child & Adolescent Psychiatry. 2016;25(8):843-52.
24. Briskman J, Castle J, Blackeby K, Bengo C, Slack K, Stebbens C, et al. Randomised controlled trial of the fostering changes programme. London: National Academy for Parenting Research/London: King's College/Department of Education. 2012.
25. Lang JM, Campbell K, Shanley P, Crusto CA, Connell CM. Building Capacity for Trauma-Informed Care in the Child Welfare System:Initial Results of a Statewide Implementation. Child Maltreatment. 2016;21(2):113-24.
26. Taussig HN, Culhane SE. Impact of a Mentoring and Skills Group Program on Mental Health Outcomes for Maltreated Children in Foster CareMentoring/Skills Group Program and Foster Care. JAMA Pediatrics. 2010;164(8):739-46.
27. Bellamy JL, Gopalan G, Traube DE. A national study of the impact of outpatient mental health services for children in long-term foster care. Clinical Child Psychology and Psychiatry. 2010;15(4):467-79.
28. Aarons GA, Sklar M, Mustanski B, Benbow N, Brown CH. "Scaling-out" evidence-based interventions to new populations or new health care delivery systems. Implementation Science. 2017;12(1):111.
29. Westermark PK, Hansson K, Olsson M. Multidimensional treatment foster care (MTFC): results from an independent replication. Journal of Family Therapy. 2011;33(1):20-41.
30. Petticrew M, Knai C, Thomas J, Rehfuess EA, Noyes J, Gerhardus A, et al. Implications of a complexity perspective for systematic reviews and guideline development in health decision making. BMJ Global Health. 2019;4(Suppl 1):e000899.
31. Booth A, Moore G, Flemming K, Garside R, Rollins N, Tunçalp Ö, et al. Taking account of context in systematic reviews and guidelines considering a complexity perspective. BMJ Global Health. 2019;4(Suppl 1):e000840.
32. Moher D, Shamseer L, Clarke M, Ghersi D, Liberati A, Petticrew M, et al. Preferred reporting items for systematic review and meta-analysis protocols (PRISMA-P) 2015 statement. Syst Rev. 2015;4(1):1.
33. Moher D, Liberati A, Tetzlaff J, Altman DG. Preferred reporting items for systematic reviews and meta-analyses: the PRISMA statement. PLoS medicine. 2009;6(7):e1000097.
34. Tong A, Flemming K, McInnes E, Oliver S, Craig J. Enhancing transparency in reporting the synthesis of qualitative research: ENTREQ. BMC Medical Research Methodology. 2012;12(1):181.
35. Flemming K, Booth A, Hannes K, Cargo M, Noyes J. Cochrane Qualitative and Implementation Methods Group guidance series-paper 6: reporting guidelines for qualitative, implementation, and process evaluation evidence syntheses. J Clin Epidemiol. 2018;97:79-85.
36. Harris JL, Booth A, Cargo M, Hannes K, Harden A, Flemming K, et al. Cochrane Qualitative and Implementation Methods Group guidance series-paper 2: methods for question formulation, searching, and protocol development for qualitative evidence synthesis. J Clin Epidemiol. 2018;97:39-48.
37. Booth A, Harris J, Croot E, Springett J, Campbell F, Wilkins E. Towards a methodology for cluster searching to provide conceptual and contextual "richness" for systematic reviews of complex interventions: case study (CLUSTER). BMC Medical Research Methodology. 2013;13(1):118.

For peer review only

38. UNICEF. The United Nations Convention on the Rights of the Child. London: UNICEF; 1989.
39. Arksey H, O'Malley L. Scoping studies: towards a methodological framework. International journal of social research methodology. 2005;8(1):19-32.
40. Levac D, Colquhoun H, O'Brien KK. Scoping studies: advancing the methodology. Implementation science. 2010;5(1):69.
41. Pfadenhauer LM, Gerhardus A, Mozygemba K, Lysdahl KB, Booth A, Hofmann B, et al. Making sense of complexity in context and implementation: the Context and Implementation of Complex Interventions (CICI) framework. Implementation Science. 2017;12(1):21.
42. Bonell C, Hinds K, Dickson K, Thomas J, Fletcher A, Murphy S, et al. What is positive youth development and how might it reduce substance use and violence? A systematic review and synthesis of theoretical literature. BMC public health. 2015;16(1):135.
43. Higgins JP, Green S. Cochrane handbook for systematic reviews of interventions: John Wiley & Sons; 2011.
44. Rees R, Oliver K, Woodman J, Thomas J. The views of young children in the UK about obesity, body size, shape and weight: a systematic review. BMC Public Health. 2011;11:188.
45. Shepherd J, Harden A, Rees R, Brunton G, Garcia J, Oliver S, et al. Young people and healthy eating: a systematic review of research on barriers and facilitators. Health Education Research. 2005;21(2):239-57.
46. Drummond MF, Sculpher MJ, Claxton K, Stoodart GL, Torrance G. Methods for the Economic Evaluation of Health Care Programmes. USA: Oxford University Press; 2015.
47. Vergnes J-N, Marchal-Sixou C, Nabet C, Maret D, Hamel O. Ethics in systematic reviews. Journal of Medical Ethics. 2010;36(12):771-4.
48. Moore G, Audrey S, Barker M, Bond L, Bonell C, Hardeman W, et al. Process evaluation of complex interventions. UK Medical Research Council (MRC) guidance. 2014:1-133.
49. Oliver SR, Rees RW, Clarke-Jones L, Milne R, Oakley AR, Gabbay J, et al. A multidimensional conceptual framework for analysing public involvement in health services research. Health expectations : an international journal of public participation in health care and health policy. 2008;11(1):72-84.
50. Brunton G, Oliver S, Oliver S, Lorenc T. A synthesis of research addressing children's, young people's and parents' views of walking and cycling for transport. London: Eppi Centre; 2006.
51. Barnett-Page E, Thomas J. Methods for the synthesis of qualitative research: a critical review. BMC Medical Research Methodology. 2009;9(1):59.
52. Gale NK, Heath G, Cameron E, Rashid S, Redwood S. Using the framework method for the analysis of qualitative data in multi-disciplinary health research. BMC medical research methodology. 2013;13:117-.
53. O'Neill J, Tabish H, Welch V, Petticrew M, Pottie K, Clarke M, et al. Applying an equity lens to interventions: using PROGRESS ensures consideration of socially stratifying factors to illuminate inequities in health. J Clin Epidemiol. 2014;67(1):56-64.
54. Welch V, Petticrew M, Petkovic J, Moher D, Waters E, White H, et al. Extending the PRISMA statement to equity-focused systematic reviews (PRISMA-E 2012): explanation and elaboration. Journal of Clinical Epidemiology. 2016;70:68-89.
55. Zorzela L, Loke YK, Ioannidis JP, Golder S, Santaguida P, Altman DG, et al. PRISMA harms checklist: improving harms reporting in systematic reviews. BMJ. 2016;352.
56. Noyes J, Booth A, Moore G, Flemming K, Tunçalp Ö, Shakibazadeh E. Synthesising quantitative and qualitative evidence to inform guidelines on complex interventions: clarifying the purposes, designs and outlining some methods. BMJ Global Health. 2019;4(Suppl 1):e000893.
57. Hong QN, Pluye P, Bujold M, Wassef M. Convergent and sequential synthesis designs: implications for conducting and reporting systematic reviews of qualitative and quantitative evidence. Syst Rev. 2017;6(1):61-.

For peer review only

58. Munabi-Babigumira S, Glenton C, Lewin S, Fretheim A, Nabudere H. Factors that influence the provision of intrapartum and postnatal care by skilled birth attendants in low-and middle-income countries: a qualitative evidence synthesis. Cochrane Database of Systematic Reviews. 2017(11).
59. Balshem H, Helfand M, Schunemann HJ, Oxman AD, Kunz R, Brozek J, et al. GRADE guidelines: 3. Rating the quality of evidence. J Clin Epidemiol. 2011;64(4):401-6.
60. Lewin S, Booth A, Glenton C, Munthe-Kaas H, Rashidian A, Wainwright M, et al. Applying GRADE-CERQual to qualitative evidence synthesis findings: introduction to the series. Implementation Science. 2018;13(1):2.
61. Rehfuess EA, Booth A, Brereton L, Burns J, Gerhardus A, Mozygemba K, et al. Towards a taxonomy of logic models in systematic reviews and health technology assessments: A priori, staged, and iterative approaches. Research synthesis methods. 2018;9(1):13-24.
62. Evans RE, Craig P, Hoddinott P, Littlecott H, Moore L, Murphy S, et al. When and how do 'effective' interventions need to be adapted and/or re-evaluated in new contexts? The need for guidance. Journal of Epidemiology and Community Health. 2019;73(6):481-2.
63. Craig P, Dieppe P, Macintyre S, Michie S, Nazareth I, Petticrew M, et al. Developing and evaluating complex interventions: the new Medical Research Council guidance. BMJ (Clinical research ed). 2008;337:a1655-a.

For peer review only

Authors' Contributions

RE secured funding for the study. RE, SM, HM, JN, MR, GMT, RT and SW contributed to the design of the systematic review. SW and HM developed the search strategy. MB and CW advised on the PPI engagement and stakeholder consultation strategy. RE drafted the protocol. All authors read and approved the final manuscript. RE is the guarantor of the review.

Funding

This work was supported by the NIHR-PHR grant number NIHR129113.

Competing Interests

None declared.

For peer review only

Figure 1. CHIMES Review Results-based Convergent Synthesis Design

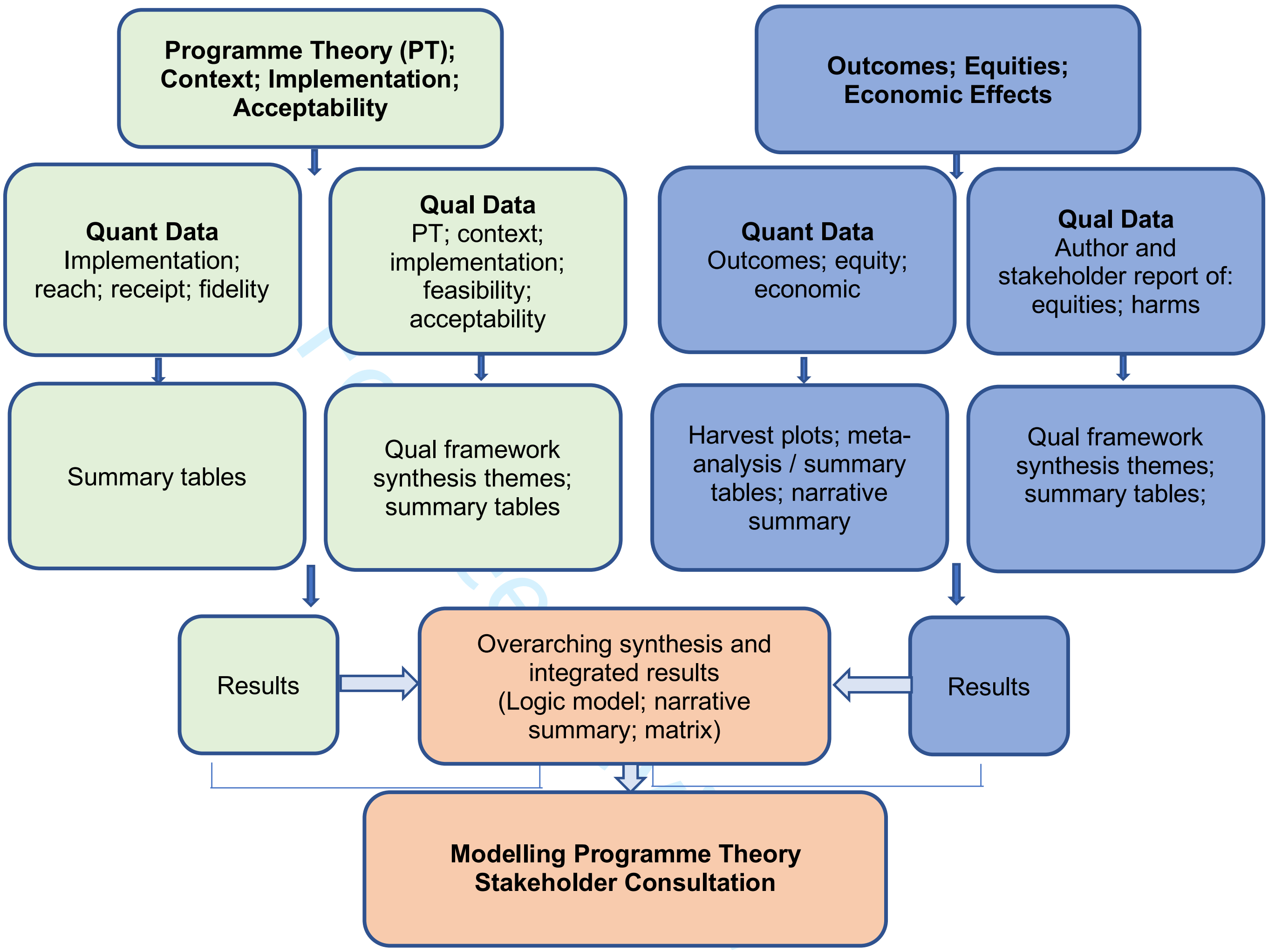

Figure 2. Intervention Prioritisation for Development, Adaptation and Evaluation in UK Context

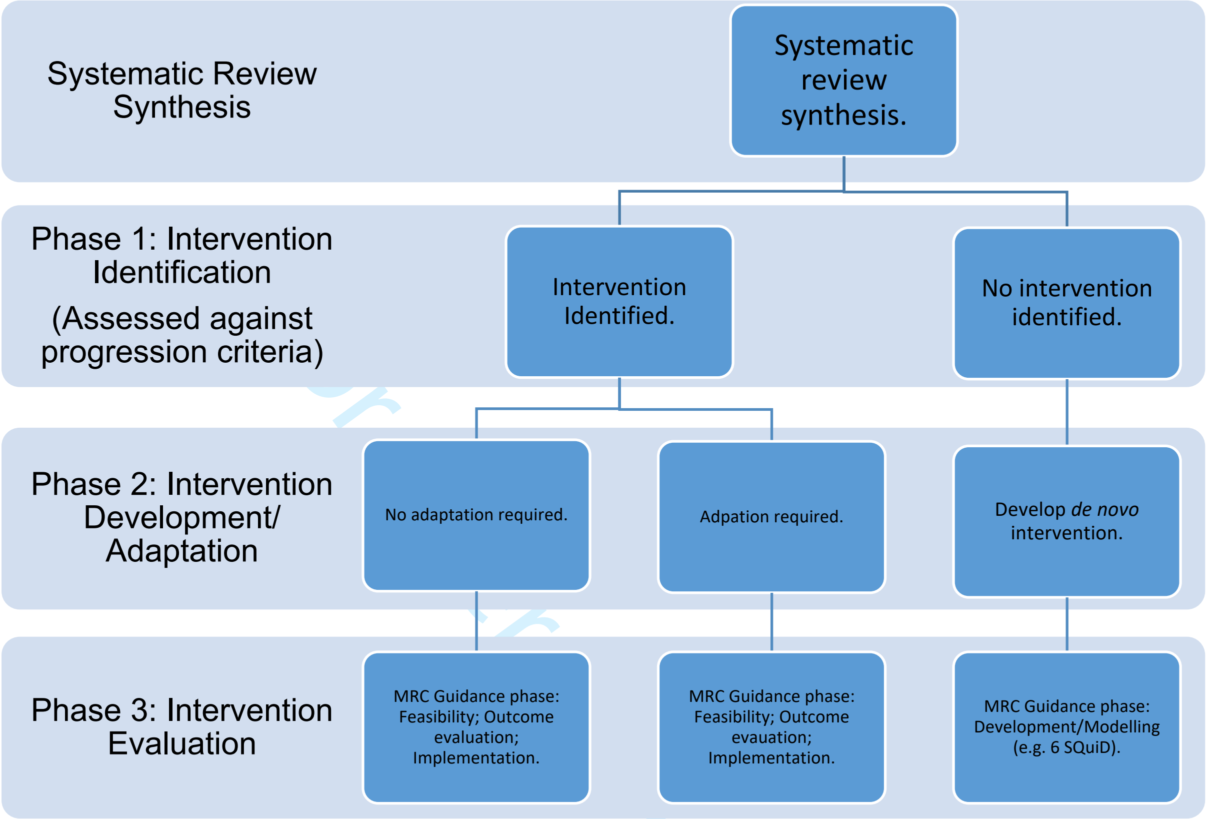

For peer review only

Supplement A: CHIMES Review Logic Model

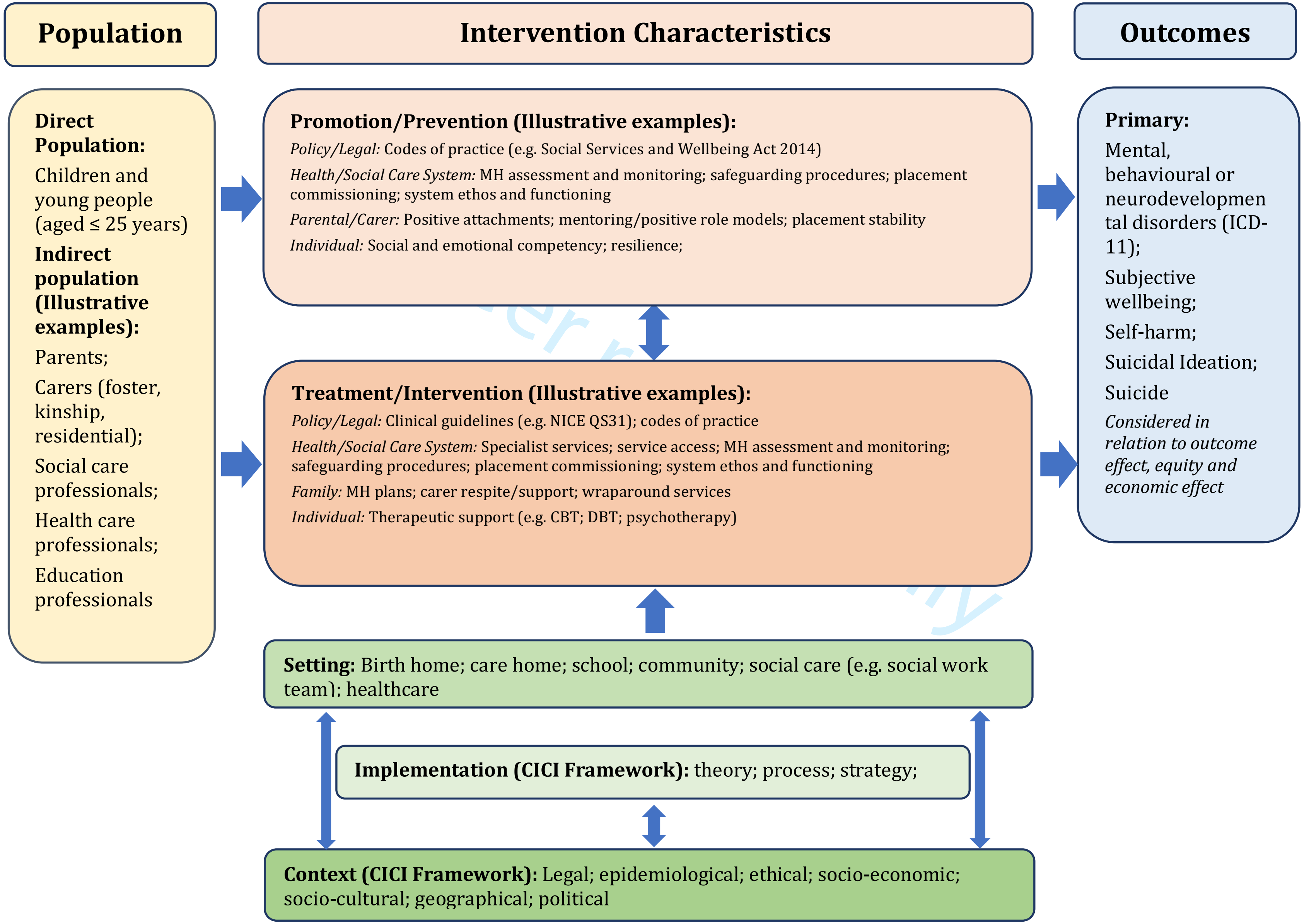

Supplement B: CHIMES Review Search Strategy (Developed in Ovid MEDLINE)

1 exp Child/
2 exp Infant/
3 Young Adult/
4 Adolescent/
5 (teen or teens or teenager*).tw.
6 (adolesc* or preadolesc* or pre-adolesc* or juvenile*).tw.
7 (youth or youths or youngster*).tw.
8 ((young adj (person or persons or people)) or "early adult*").tw.
9 (student or students or schoolchild*).tw.
10 (girl* or boy* or child or children or infant or infants or kid or kids).tw.
11 (pediatri* or paediatric*).tw.
12 (pubescen* or puberty or prepubescen* or pre-pubescen*).tw.
13 orphan*.tw.
14 Child, Foster/
15 Child, Orphaned/
16 "Child of Impaired Parents"/
17 1 or 2 or 3 or 4 or 5 or 6 or 7 or 8 or 9 or 10 or 11 or 12 or 13 or 14 or 15 or 16
18 ((substitute or "local authority" or state or statutory or public or "out of home" or order or place* or group*) adj (care or placement*)).tw.
19 ((nonparent or non-parent) adj3 care).tw.
20 ((children's or childrens) adj home).tw.
21 ((institution* or residential or foster or kinship or group) adj3 (care or home* or placement*)).tw.
22 ("support* living" or "supported lodging*" or "care leaver*").tw.
23 (leaving adj2 care).tw.
24 ((in or welfare or social or respite) adj care).tw.
25 looked after.tw.
26 Special guardian*.tw.
27 Foster Home Care/
28 Child, Institutionalized/
29 18 or 19 or 20 or 21 or 22 or 23 or 24 or 25 or 26 or 27 or 28
30 "Quality of Life"/
31 personal satisfaction/
32 (wellbeing or well-being or "well being").tw.
33 (illbeing or ill-being or "ill being").tw.
34 hedoni*.tw.
35 (eudaimoni* or eudaemoni* or eudemoni*).tw.
36 happiness.tw.
37 ((positive or negative) adj affect).tw.
38 flourish*.tw.
39 ("life satisfaction" or "satisfaction with life").tw.
40 ("positive and negative affect schedule" or PANAS).tw.
41 ("Warwick-Edinburgh Mental Wellbeing" or WEMWBS).tw.
42 ("State Trait Anxiety Inventory" or STAI).tw.
43 "Perceived Stress Scale".tw.
44 SWLS.tw.
45 30 or 31 or 32 or 33 or 34 or 35 or 36 or 37 or 38 or 39 or 40 or 41 or 42 or 43 or 44
46 Mental Health/
47 exp Mental Disorders/
48 Catatonia/
49 Self-Injurious Behavior/
50 Self Mutilation/

For peer review only

51 Suicide/
52 Suicidal Ideation/
53 Suicide, Attempted/
54 Suicide, Completed/
55 "mental health".tw.
56 ("bodily distress" or paraphilic or paraphilia or catatonia or catatonic or dissociation or "impulse control").tw.
57 (schizophrenia or psychotic or psychosis or OCD or "obsessive compulsive disorder").tw.
58 suicid*.tw.
59 (self adj2 (harm or injur* or cutting or mutilation or poison* or burn*)).tw.
60 (("post traumatic" or post-traumatic or posttraumatic) adj2 (stress or disorder*)).tw.
61 ((grief or adjustment or "reactive attachment" or "disinhibited social engagement") adj2 (disorder* or condition* or problem*)).tw.
62 (disruptive adj2 behavio?r*).tw.
63 ((behavio?r* or neurodevelopmental or mood or fear or anxiety or personality or disruptive or dissocial or impulse or factitious or neurocognitive or feeding or eating or elimination or disruptive or dissocial or anxiety or depressive) adj3 (disorder* or condition* or problem*)).tw.
64 46 or 47 or 48 or 49 or 50 or 51 or 52 or 53 or 54 or 55 or 56 or 57 or 58 or 59 or 60 or 61 or 62 or 63
65 45 or 64
66 exp Controlled Clinical Trial/
67 Double-Blind Method/
68 exp "Costs and Cost Analysis"/
69 random allocation/
70 (("pre-test" or pretest or posttest or "post-test" or "pre-intervention" or "post-intervention" or "controlled before" or "before and after" or "follow-up assessment") and (controlled or control or "comparison participants" or "comparison group" or "usual care" or placebo)).tw.
71 ("quasi-experiment*" or quasiexperiment* or "quasi-randomi*" or "quasirandomi*" or "natural* experiment" or "time series" or "interrupted time").tw.
72 ((controlled or control or intervention or comparison) adj3 (group or groups or study or trial or evaluation or cohort or cohorts or longitudinal or matched or matching or experiment or experimental)).tw.
73 ("difference in difference" or "instrumental variable*" or "propensity score matching" or "process evaluation").tw.
74 ((cost or costs or costing or economic) adj1 (analysis or effectiveness or benefit or evaluation or utility or savings or measure or measures)).tw.
75 (trial or "randomi?ed controlled trial" or rct or "cross-over design" or " cross over design" or "crossover design" or "cross-over study" or "cross over study" or "crossover study" or "factorial design" or "controlled study" or "controlled design" or "single-blind" or "single blind" or "double-blind" or "double blind" or "triple-blind" or "triple blind").tw.
76 effectiveness.tw.
77 program evaluation/
78 (program* adj (effect* or efficacy)).tw.
79 ((theor* or mechanism*) adj3 (change or impact or program*)).tw.
80 (("mixed method*" or "mixed-method*" or process or qualitative) adj3 evaluation*).tw.
81 (causal adj2 (assumption* or process*)).tw.
82 66 or 67 or 68 or 69 or 70 or 71 or 72 or 73 or 74 or 75 or 76 or 77 or 78 or 79 or 80 or 81
83 17 and 29 and 65 and 82

PRISMA-P (Preferred Reporting Items for Systematic review and Meta-Analysis Protocols) 2015 checklist: recommended items to address in a systematic review protocol*

Section and topic	Item No	Checklist item	Location in Text
**ADMINISTRATIVE INFORMATION**			
Title:			
Identification	1a	Identify the report as a protocol of a systematic review	p2
Update	1b	If the protocol is for an update of a previous systematic review, identify as such	N/A
Registration	2	If registered, provide the name of the registry (such as PROSPERO) and registration number	p2
Authors:			
Contact	3a	Provide name, institutional affiliation, e-mail address of all protocol authors; provide physical mailing address of corresponding author	p1
Contributions	3b	Describe contributions of protocol authors and identify the guarantor of the review	p19
Amendments	4	If the protocol represents an amendment of a previously completed or published protocol, identify as such and list changes; otherwise, state plan for documenting important protocol amendments	N/A
Support:			
Sources	5a	Indicate sources of financial or other support for the review	p19
Sponsor	5b	Provide name for the review funder and/or sponsor	p19
Role of sponsor or funder	5c	Describe roles of funder(s), sponsor(s), and/or institution(s), if any, in developing the protocol	p19
**INTRODUCTION**			
Rationale	6	Describe the rationale for the review in the context of what is already known	pp. 4-5
Objectives	7	Provide an explicit statement of the question(s) the review will address with reference to participants, interventions, comparators, and outcomes (PICO)	pp. 5-6
**METHODS**			
Eligibility criteria	8	Specify the study characteristics (such as PICO, study design, setting, time frame) and report characteristics (such as years considered, language, publication status) to be used as criteria for eligibility for the review	pp. 6-7
Information sources	9	Describe all intended information sources (such as electronic databases, contact with study authors, trial registers or other grey literature sources) with planned dates of coverage	p7
Search strategy	10	Present draft of search strategy to be used for at least one electronic database, including	p7 /Supplement B

For peer review only

		planned limits, such that it could be repeated	
Study records:			
Data management	11a	Describe the mechanism(s) that will be used to manage records and data throughout the review	p8
Selection process	11b	State the process that will be used for selecting studies (such as two independent reviewers) through each phase of the review (that is, screening, eligibility and inclusion in meta-analysis)	p8
Data collection process	11c	Describe planned method of extracting data from reports (such as piloting forms, done independently, in duplicate), any processes for obtaining and confirming data from investigators	p8
Data items	12	List and define all variables for which data will be sought (such as PICO items, funding sources), any pre-planned data assumptions and simplifications	pp.8-9
Outcomes and prioritization	13	List and define all outcomes for which data will be sought, including prioritization of main and additional outcomes, with rationale	pp.6-7/ Supplement A
Risk of bias in individual studies	14	Describe anticipated methods for assessing risk of bias of individual studies, including whether this will be done at the outcome or study level, or both; state how this information will be used in data synthesis	p9
Data synthesis	15a	Describe criteria under which study data will be quantitatively synthesised	pp.9-11
	15b	If data are appropriate for quantitative synthesis, describe planned summary measures, methods of handling data and methods of combining data from studies, including any planned exploration of consistency (such as I^2, Kendall's τ)	pp.9-11
	15c	Describe any proposed additional analyses (such as sensitivity or subgroup analyses, meta-regression)	pp.9-11
	15d	If quantitative synthesis is not appropriate, describe the type of summary planned	
Meta-bias(es)	16	Specify any planned assessment of meta-bias(es) (such as publication bias across studies, selective reporting within studies)	
Confidence in cumulative evidence	17	Describe how the strength of the body of evidence will be assessed (such as GRADE)	p11

***It is strongly recommended that this checklist be read in conjunction with the PRISMA-P Explanation and Elaboration (cite when available) for important clarification on the items. Amendments to a review protocol should be tracked and dated.** **The copyright for PRISMA-P (including checklist) is held by the PRISMA-P Group and is distributed under a Creative Commons Attribution Licence 4.0.**

From: Shamseer L, Moher D, Clarke M, Ghersi D, Liberati A, Petticrew M, Shekelle P, Stewart L, PRISMA-P Group. Preferred reporting items for systematic review and meta-analysis protocols (PRISMA-P) 2015: elaboration and explanation. BMJ. 2015 Jan 2;349(jan02 1):g7647.

For peer review only
